# Description of the skull, braincase, and dentition of *Moschognathus whaitsi* (Dinocephalia, Tapinocephalia), and its palaeobiological and behavioral implications

**DOI:** 10.1002/ar.70038

**Published:** 2025-09-08

**Authors:** Tristen Lafferty, Luke A. Norton, Aliénor Duhamel, Julien Benoit

**Affiliations:** ^1^ Evolutionary Studies Institute University of the Witwatersrand Johannesburg South Africa; ^2^ School of Geosciences University of the Witwatersrand Johannesburg South Africa

**Keywords:** braincase, CT‐scanning, Dinocephalia, headbutting, pathology, tooth replacement

## Abstract

A subadult *Moschognathus whaitsi* from the Eastern Cape Province, South Africa, was scanned using synchrotron radiation X‐ray computed tomography (SRXCT). Its subadult state allowed the cranial bones and teeth to be identified and individually reconstructed in 3D. A complete description of every preserved cranial bone is here produced, with special attention given to the braincase. An evaluation of a frontal abscess has provided insight into the potential behavior of *Moschognathus*, regarding the long‐standing hypothesis of dinocephalian headbutting. The first 3D description of tapinocephalian dentition shows the unique talon‐and‐heel tooth morphology and expands our understanding of the replacement patterns in the dentition of basal therapsids. We report evidence of at least three successive replacement generations developing in the incisiform dentition of *M. whaitsi* simultaneously. The arrangement of these successive generations of replacement incisiform teeth is reminiscent of the complex dental batteries described in sauropod dinosaurs. Furthermore, an alternating pattern of replacement is apparent and suggests that the upper and lower rows of functional teeth were comprised of at least two tooth generations.

## 1 INTRODUCTION

Dinocephalians are a middle Permian suborder of the clade Therapsida. Therapsids are often defined as the paraphyletic stem group of Mammalia (Kemp, 2006) and are divided into five monophyletic and one paraphyletic group: the Biarmosuchia, Anomodontia, Dinocephalia, Gorgonopsia, Therocephalia, and Cynodontia, respectively. Dinocephalians from southern Africa can be divided into the carnivorous Anteosauria and the primarily herbivorous Tapinocephalia (Fraser‐King et al., 2019; Kammerer, 2011). Both clades went extinct during the end‐Capitanian extinction event (~260 MA; see Day and Rubidge, 2021).

In contrast to other therapsid groups, in‐depth studies of dinocephalian braincases and cranial endocasts are scarce (Benoit, Fernandez, et al., 2017; Castanhinha et al., 2013; Duhamel et al., 2024; Pusch et al., 2019, 2020, 2024; Rodrigues et al., 2014). Skull thickness, nervous system anatomy, and head orientation have been studied in only a handful of taxa (Barghusen, 1975; Benoit, Kruger, et al., 2021; Benoit, Manger, et al., 2017; Benoit & Midzuk, 2024; Boonstra, 1968). In 1968, L.D. Boonstra published a descriptive paper on the internal cranial anatomy in several genera of dinocephalians, including *Struthiocephalus whaitsi*, *Keratocephalus moloch*, *Moschops capensis*, *Anteosaurus magnificus*, and *Jonkeria truculenta*. These descriptions were obtained via mechanical, destructive means: using either a diamond‐studded circular saw to cut serial sections or hammer and chisels to induce cracks to expose the internal anatomy (Boonstra, 1968). The skull and braincase of *Ulemosaurus*, a Russian Tapinocephalidae, have sometimes been displayed in the sagittal section (Tatarinov, 1976) and are characterized by a shortening of the paroccipital process, hypothesized to have resulted from the necessity of retaining a consistent braincase volume (Ivakhnenko, 2008).

Benoit, Manger, et al. (2017) described the endocast, bony labyrinth, and trigeminal nerve of a subadult specimen (AM 4950) previously identified as *M. capensis*. Benoit, Manger, et al. (2017) calculated the encephalization quotient of AM 4950; their results showed that AM 4950 had a higher encephalization quotient than expected for a Permian herbivore. This research was followed up when Benoit and Midzuk (2024) calculated the encephalization quotients of *Anteosaurus* and *Jonkeria*. These results further showed both dinocephalians to have a higher encephalization quotient than other non‐mammalian therapsids (Benoit & Midzuk, 2024). Apart from Benoit, Manger, et al. (2017); Benoit, Kruger, et al. (2021); and Benoit and Midzuk (2024), work on dinocephalians has been limited to two‐dimensional analysis. Here we provide the first comprehensive description of the cranium and dentition of a specimen assigned to *Moschognathus whaitsi*, based on three‐dimensional (3D) SRXCT imagery.

### 1.1 Institutional abbreviations

AM—Albany Museum, Makhanda, South Africa

AMNH—American Museum of Natural History, New York, NY, USA

ESRF—European Synchrotron Radiation Facility, Grenoble, France

NCSM—North Carolina Museum of Natural History, Raleigh, NC, USA

SAM—Iziko South African Museum, Cape Town, South Africa

## 2 MATERIALS AND METHODS

### 2.1 Fossil material

**FIGURE 1:**
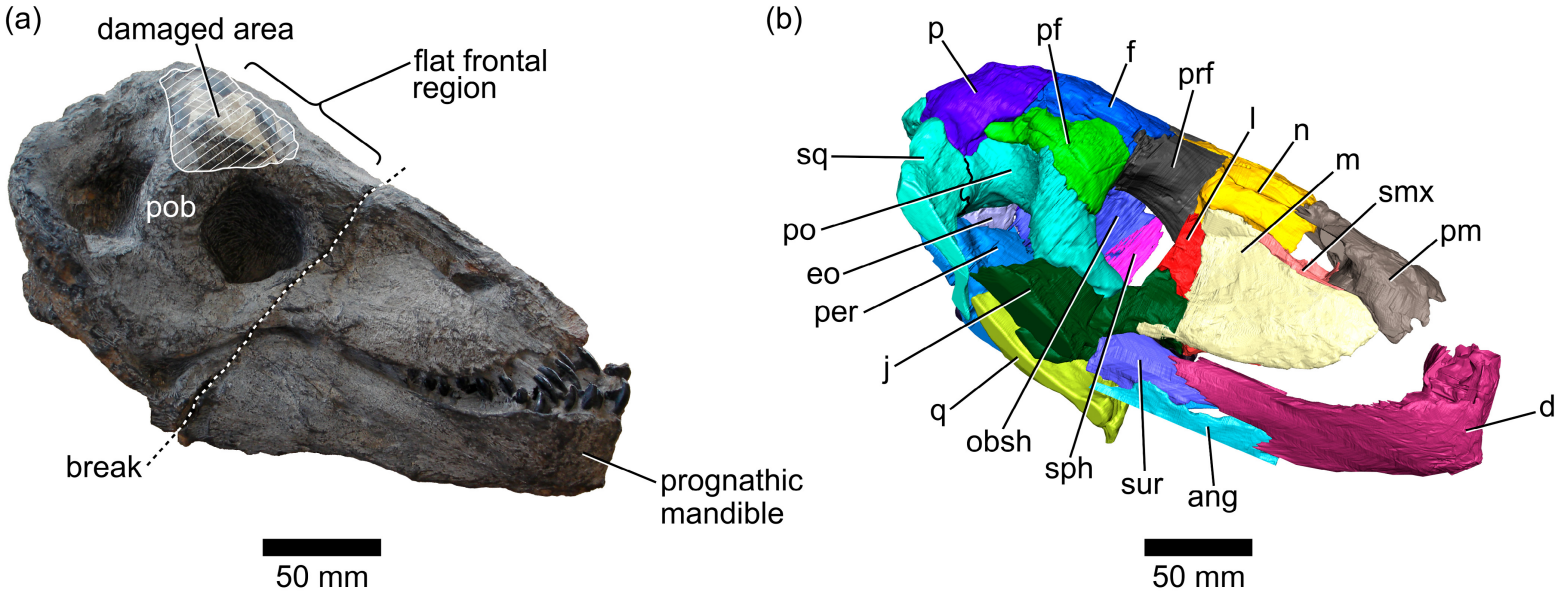
Skull of *Moschognathus whaitsi* (AM 4950) in lateral view. Photograph (a) and 3D surface model (b). Break between anterior and posterior portions indicated by a dashed line, and prefrontal and frontal regions damaged during preparation shown with hatching. ang, angular; d, dentary; eo, exoccipital; f, frontal; j, jugal; l, lacrimal; m, maxilla; n, nasal; obsh, orbitosphenoid; p, parietal; par, paroccipital; pf, postfrontal; pm, premaxilla; po, postorbital; pob, postorbital bar; prf, prefrontal; q, quadrate; smx, septomaxilla; sph, sphenethmoid; sq, squamosal; sur, surangular. Scale bars equal 50 mm.

**FIGURE 2:**
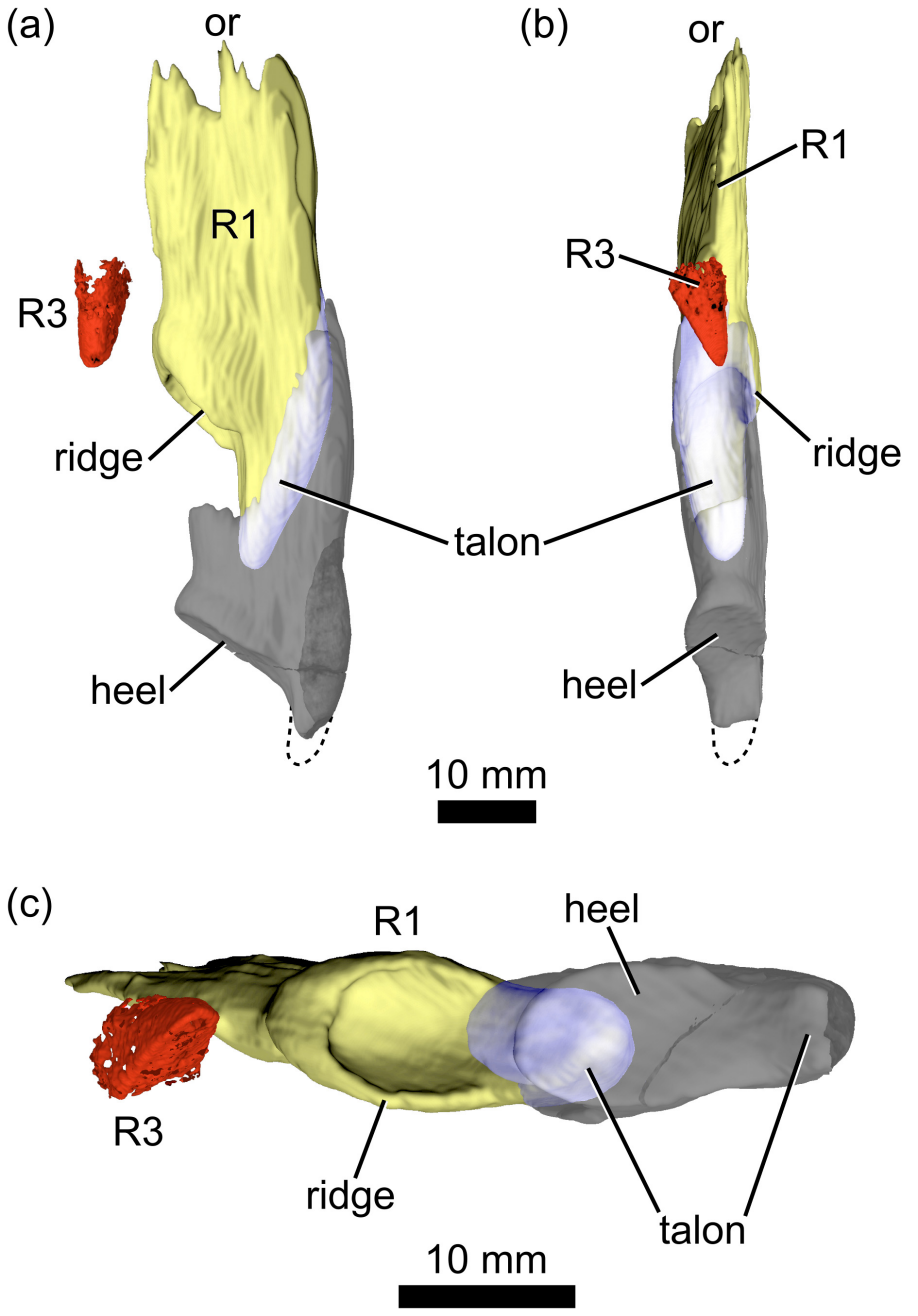
Isolated tooth family of fourth upper incisiform (I4) of *Moschognathus whaitsi* (AM 4950). Lateral (a), lingual (b) and occlusal (c) views. Note how the root of the functional tooth (semi‐transparent gray) has been resorbed, allowing the talon of the primary replacement (R1, yellow) to pierce the pulp cavity, whereas the developing tertiary replacement (R3, vermillion) is positioned some distance from R1. Dotted lines indicate reconstruction of damaged talon of the functional tooth. or, open root; R1, primary replacement, R3, tertiary replacement. Scale bars equal 10 mm.

Specimen AM 4950 is a well‐preserved subadult *M. whaitsi* skull (Figure 1a), stored in the collection of the Albany Museum in Makhanda (formerly Grahamstown), South Africa. It was collected from a farm named The Grant 39 in the Sarah Baartman District, Eastern Cape Province, South Africa (Mason et al., 2015). The farm is located north of Makhanda, and its rock exposures are assigned to the *Diictodon‐Styracocephalus* Subzone of the *Tapinocephalus* Assemblage Zone (late Permian, Wordian, ~265 Ma; Day and Rubidge, 2020). The specimen is broken into two halves, the snout and the postorbital regions (Figure 1a). Initially identified as a juvenile anteosaurid and tentatively assigned to the genus *Anteosaurus* by Modesto et al. (2001), preparation of AM 4950 revealed the distinctive talon‐and‐heel dental morphology typical of tapinocephalid dinocephalians (Figure 2). It was subsequently identified as *Moschops* (Benoit, Manger, et al., 2017) and later reclassified as a subadult *Moschognathus* (Benoit, Kruger, et al., 2021; Neumann, 2020). Neumann (2020) reclassified AM 4950 as a subadult *Moschognathus* based on its longer snout and less pachyostosis than other similar‐sized *Moschops*.

### 2.2 Synchrotron scanning and data availability

SRXCT scanning of the specimen was performed at the ESRF in Grenoble, France. The anterior and posterior regions of AM 4950 were scanned separately with isotropic voxel sizes of 91 and 117.23 μm, respectively (see Benoit, Manger, et al., 2017 for full details regarding the scanning methods and parameters used). The data supporting the findings of this study are available through the ESRF's Paleontology Database at http://doi.org/10.15151/ESRF-DC-1634315861.

### 2.3 Segmentation using Avizo

The 3D reconstruction of the cranium and lower jaw was performed manually at the Virtual Image Processing Laboratory at the Evolutionary Studies Institute (ESI), University of the Witwatersrand, using Avizo 2020 (FEI VSG, Hillsboro, OR, USA). Segmentation was done by manually selecting the desired elements every 10 slides and using the interpolation function. This process was repeated for each bone until every cranial bone was segmented. Once each bone was segmented, photos and measurements were taken as the basis for the descriptive work.

### 2.4 Segmentation using VG Studio MAX

The dentition was segmented using VG Studio MAX 3.2.4 (Volume Graphics, Heidelberg, Germany). Each tooth was manually segmented as a separate region of interest (ROI). Every second slice was segmented using the “polygon lasso” and “polyline” tools. The intermediate slices were then filled using the “open/close” function, by first expanding the selected ROI by two voxels, followed by shrinking the ROI by one voxel. Replacement teeth were recognizable due to the presence of mineralized tissue and are therefore considered to be at least in an advanced bell stage of development (Luckett, 1993).

### 2.5 Tooth analysis

Most previous studies of therapsid tooth replacement using CT have reported only a single replacement tooth associated with each tooth position (Abdala et al., 2013; Norton, 2020; Norton et al., 2020, 2021; Olroyd et al., 2021). Boonstra (1962) was the first to describe evidence for multiple replacement generations lingual to the functional dentition, as in many groups of dinosaurs (D'Emic et al., 2013, 2024; He et al., 2018). As a consequence, we use a modified notation to that proposed by Smith and Dodson (2003) for non‐mammalian tetrapods. Rather than differentiating between teeth based on which bone they are socketed in, we have used the variation in crown morphology and tooth size to distinguish between an anterior “incisiform” (I/i) and posterior “postcanine” (PC/pc) dentition. Uppercase abbreviations are used when referring to upper dentition (i.e., I4 refers to the fourth incisiform tooth of the upper series), whereas lowercase abbreviations are used for the lower dentition (i.e., pc6 refers to the sixth postcanine of the lower series). Furthermore, we expand upon the color scheme of Norton (2020) to better distinguish between replacement teeth belonging to different generations.

### 2.6 Estimating tooth replacement rates

Despite not having access to the incremental growth lines of the dentine of AM 4950 at present, the method described by Erickson (1996b) to estimate replacement rate in dentition using CT data, rather than histological sections, was adapted and applied to AM 4950. A range of daily dentine apposition rates (DDAR) for various extinct and extant taxa was compiled from the literature (Table 1). These taxa were chosen based on: (a) the polyphyodont nature of the incisiform dentition of AM 4950; (b) the hypothesis that *Moschognathus* occupied an ecological role similar to that of modern megaherbivores, and (c) the tooth morphology and potentially a feeding mechanism of AM 4950 being comparable to sauropod dinosaurs. The 21 taxa included in Table 1 comprise non‐mammalian therapsids, sauropod dinosaurs, as well as extinct and extant mammalian megaherbivores. Sauropod dinosaurs and mammalian megaherbivores were chosen based on the hypothesis that *Moschognathus* may have filled a similar ecological role during the Permian, whereas the additional non‐mammalian therapsid taxa were included due to their phylogenetic proximity to tapinocephalid dinocephalians.

**TABLE 1:** Dentine incremental line distances reported in extinct and extant taxa.

Higher taxon	Clade	Genus/species	DDAR (μm day^−1^)		Source
			Range	Average	
Synapsida	“Pelycosaur”	*Dimetrodon* ^a^	—	5.46	Maho et al. (2022)
Synapsida	Dinocephalia	Tapinocephalidae indet.^b^	20–40	34	Whitney and Sidor (2019)
Synapsida	Dicynodontia	*Diictodon*	16–22	20	Thackeray (1991)
Synapsida	Dicynodontia	*Lystrosaurus*	—	17.4	Jasinoski and Chinsamy‐Turan (2012)
Synapsida	Dicynodontia	*Lystrosaurus* (South Africa)^b^	13.58–22.92	15.21	Whitney and Sidor (2020)
Synapsida	Dicynodontia	*Lystrosaurus* (Antarctica)^b^	10.04–22.86	16.29	Whitney and Sidor (2020)
Synapsida	Dicynodontia	Dicynodontia indet. (NCSM 19585)^b^	12.50–33.33	18.79	Green (2012)
Synapsida	Dicynodontia	Dicynodontia indet. (NCSM 21735)^b^	20–50	31.50	Green (2012)
Synapsida	Cynodontia	*Diademodon*	—	11.9	O'Meara et al. (2018)
Synapsida	Cynodontia	*Cricodon metabolus* ^b^	—	22.22	Hendrickx et al. (2016)
Synapsida	Cynodontia	*Tritylodon*	13.6–13.7	13.65	Jasinoski and Chinsamy (2012)
Mammalia	Proboscidea	*Mammut* sp.^b^	—	16.16	Schour and Hoffman (1939)
Mammalia	Proboscidea	*Mammut americanum*	9.48–25.00	15.13	Koch (1989)
Mammalia	Proboscidea	*Gomphotherium*	11.71–15.86	13.37	Fox (2000)
Mammalia	Proboscidea	*Loxodonta africana*	—	15.07	Codron (2008)
Mammalia	Proboscidea	*Mammuthus* sp.	16.63–20.00	18.46	Koch (1989)
Mammalia	Proboscidea	*Palaeoloxodon falconeri*	2.00–6.94	4.10	Köhler et al. (2021)
Mammalia	Proboscidea	*Elephas maximus*	—	16	Koch (1989)
Mammalia	Pantodonta	*Coryphodon* sp.	—	21.48	Finch and D'Emic (2022)
Mammalia	Artiodactyla	*Giraffa cameolopardalis*	15–20	17.5	Nacarino‐Meneses et al. (2025)
Dinosauria	Saurischia	*Diplodocus*	—	15	D'Emic et al. (2013)
Dinosauria	Saurischia	*Camarasaurus*	—	15	D'Emic et al. (2013)
Dinosauria	Saurischia	*Abydosaurus mcintoshi*	—	9.7	Finch and D'Emic (2022)
Dinosauria	Saurischia	Titanosauria indet.	13–30	21	García and Zurriaguz (2016)

Abbreviations: DDAR, daily dentine apposition rate; indet., indeterminate.

^a^ Not included in calculations of average incremental thickness.

^b^ Considered a problematic taxon by Finch and D'Emic (2022).

An average DDAR was calculated for each group, as well as for the total sample (Table 2). Due to the wide range in values (~4–34 μm day^−1^; Table 1), it was necessary to determine whether the samples included any outliers. Potential outliers were identified using the 1.5 IQR (interquartile range) rule (Zar, 2013). Whereas Erickson (1996b) used a single representative tooth of average crown volume per specimen to estimate the DDAR, we have instead used the central tooth from the upper and lower incisiform series (i.e., I4 and i4, respectively) as the representative tooth in calculations of the DDAR.

**TABLE 2:** Average daily dentine apposition rates used to estimate tooth replacement rates of *Moschognathus whaitsi* (AM 4950).

Taxon	DDAR (μm day^−1^)	Source	Comments
Tapinocephalidae indet.	34^a^	Whitney and Sidor (2019)	Exceeds upper limit for non‐mammalian therapsids
Therapsida (*n* = 10)	20.10	Table 1	
Therapsida (*n* = 8)	16.93^a^	Table 1	Outliers removed
Mammalia (*n* = 9)	15.25	Table 1	
Mammalia (*n* = 8)	16.65^a^	Table 1	Outlier removed
Sauropoda (*n* = 4)	15.18^a^	Table 1	
All taxa (*n* = 23)	17.34	Table 1	
All taxa (*n* = 20)	16.47^a^	Table 1	Outliers removed
All Taxa	16	Schour and Hoffman (1939)	

Abbreviations: DDAR, daily dentine apposition rate; indet., indeterminate.

^a^ Used to estimate tooth replacement rate (see Table 4).

Dentine thickness was measured in silico from the border of the pulp cavity to the contact with the enamel, perpendicular to the hypothesized trajectory of the incremental lines. The difference in dentine thickness between successive teeth of the same tooth family was calculated (Table 3). These were averaged according to tooth type (incisiform or postcanine) and developmental state (functional−primary/secondary replacement, primary replacement−tertiary replacement, etc.). Together with the representative upper (I4) and lower (i4) incisiform tooth, these values were divided by the average incremental line thicknesses for each group reported in Table 2, to calculate a range of tooth replacement rates for AM 4950 (Table 4).

**TABLE 3:** Difference in dentine thickness between successive tooth generations in *Moschognathus whaitsi* (AM 4950).

Upper locus	Difference in dentine thickness (mm)		
	Func.−R1	Func.−R2	R1−R3
I1	—	4.65	—
I2	1.69^a^	—	—
I3	—	3.97	—
I4	3.40	—	2.99
I5	—	4.02	—
I6	0.34	—	2.83
I7	—	2.89	—
PC1	1.23	—	—
PC2	—	1.93	—
PC3	0.33	—	—
PC4	—	1.35	—
PC5	—	—	—
PC6	—	—^b^	—
PC7	—	—	—
PC8	—	—	—

Lower locus	func.−r1	func.−r2	r1−r3
i1	—	3.43	0.56^c^
i2	2.71	—	2.22
i3	—	2.39	—
i4	1.10	—	2.48
i5	—	2.83	—
i6	1.00	—	—
i7	—^d^	—	—
i8	3.15	—	—
pc1	—	—	—
pc2	—	—	—
pc3	—	—	—
pc4	—^e^	—	—
pc5	—	—	—
pc6	0.58	—	—
pc7	—	—	—

Abbreviations: Func., functional tooth; R1/r1, primary replacement; R2/r2, secondary replacement; R3/r3, tertiary replacement.

^a^ Labial surface of functional tooth (I2) lost due to damage.

^b^ Insufficient contrast to determine the dentine boundary of secondary replacement tooth (PC6.R2).

^c^ Successive replacements in this tooth family (i.e., i1.r2, and i1.r3) are identified as secondary and tertiary replacements, rather than primary and tertiary replacements typical of other loci where more than one developing replacement is present.

^d^ Insufficient contrast to determine the dentine boundary of functional tooth (i7).

^e^ Insufficient contrast to determine the dentine boundary of the primary replacement tooth (pc4.r1).

**TABLE 4:** Estimated tooth replacement rates for *Moschognathus whaitsi* (AM 4950).

		Average dentine thickness (mm)					Estimated replacement rate (days)^a^				
		Func.−R1	Func.−R2	R1−R3	All teeth		Tap. indet.	Therapsida	Mammalia	Sauropoda	All taxa
Representative tooth	I4	3.40	—	2.99	3.19		88–100	177–201	180–204	197–224	182–206
	i4	1.10	—	2.48	1.79		32–73	65–146	66–149	72–163	67–151
Average incisiform	Upper	1.81	3.88	2.91	2.98		53–114	107–229	109–233	119–256	110–236
	Lower	1.99	2.88	1.75	2.19		51–85	103–170	105–173	115–190	106–175
Average postcanine	Upper	0.78	1.64	—	1.21		23–48	46–97	47–98	51–108	47–100
	Lower	0.58	—	—	0.58		17	34	35	38	35

Abbreviations: DDAR, daily dentine apposition rate; Func., Functional tooth; Tap. indet., Tapinocephalidae indeterminate.

^a^ Values used correspond to the DDARs presented in Table 2.

## 3 CRANIAL DESCRIPTION

### 3.1 General remarks

Specimen AM 4950 displays typical Moschopinae (Dinocephalia: Tapinocephalia) characteristics, including thick frontal and parietal bones, large postorbital bars, a prognathic mandible, a diagnostic talon‐and‐heel morphology of the dentition, and an angled occiput (Figures 1 and 2) (Benoit, Manger, et al., 2017). What separates *Moschognathus* from other members of Moschopinae is a flatter frontal bone, a laterally narrow snout, a narrow nasal posterior process, and, in this specimen, larger orbits (possibly caused by the subadult state) (Figure 1). It should be noted that Neumann (2020) did not list any autapomorphies for *Moschognathus*. However, past researchers have also used a dorsally flat skull and narrow snout as distinct characters that separate *Moschognathus* from *Moschops* (Broom, 1932; Gregory, 1926; Neumann, 2020). There appear to be little to no interdigitating sutures in the cranium of AM 4950, as most cranial sutures are relatively simple.

**FIGURE 3:**
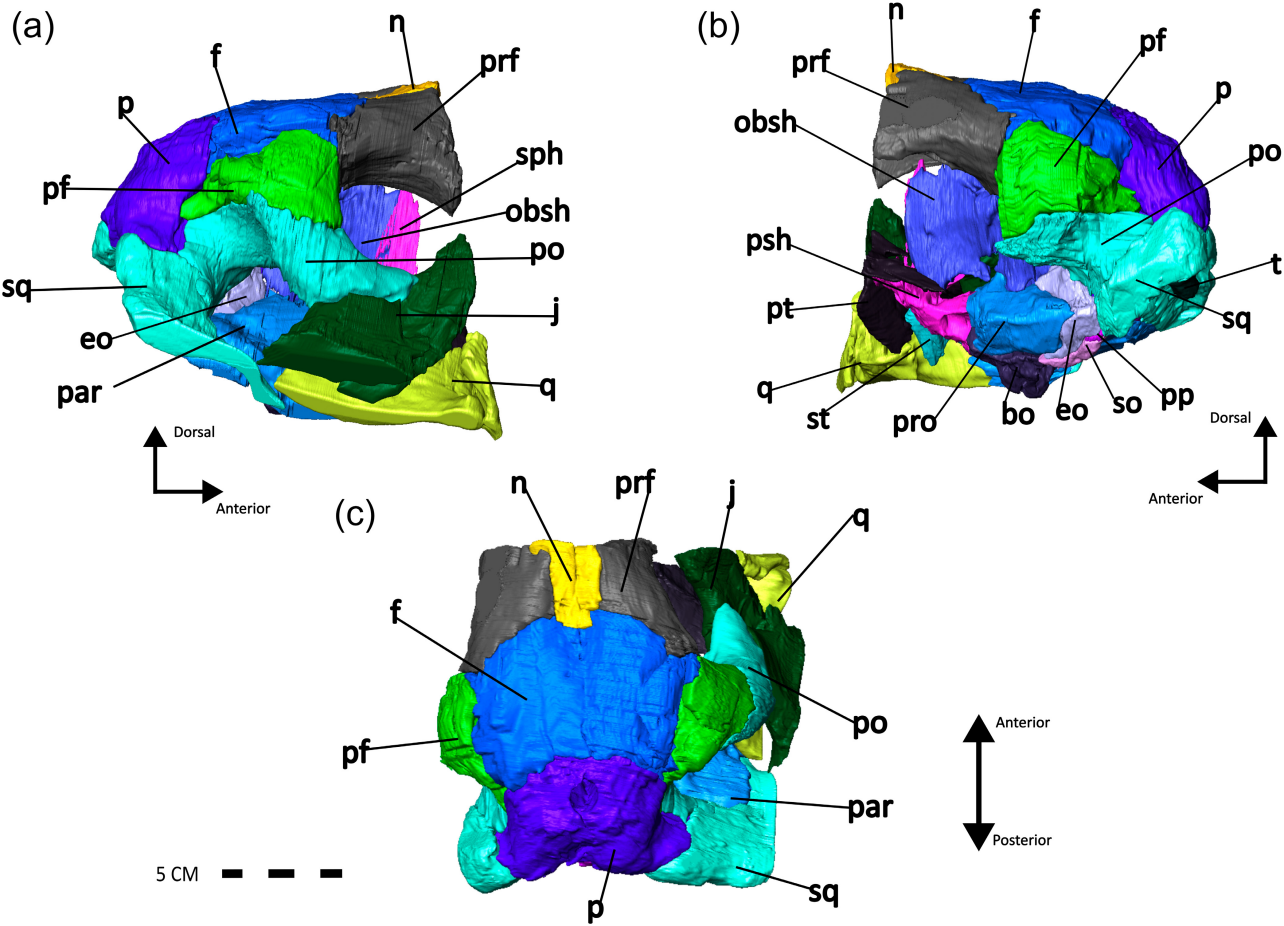
*Moschognathus whaitsi* (Capitanian, South Africa), juvenile specimen of AM 4950, posterior portion of skull. (a) Right lateral view. (b) Left lateral view. (c) Dorsal view. bo, basioccipital; eo, exoccipital; f, frontal; j, jugal; n, nasal; obsh, orbitosphenoid; p, parietal; par, paroccipital; prf, prefrontal; pf, postfrontal; pp, postparietal; pro, prootic; po, postorbital; psh, parasphenoid; pt, pterygoid; q, quadrate; sph, sphenethmoid; sq, squamosal; st, stapes; so, supraoccipital; t, tabular. Scale bar equals 5 cm.

**FIGURE 4:**
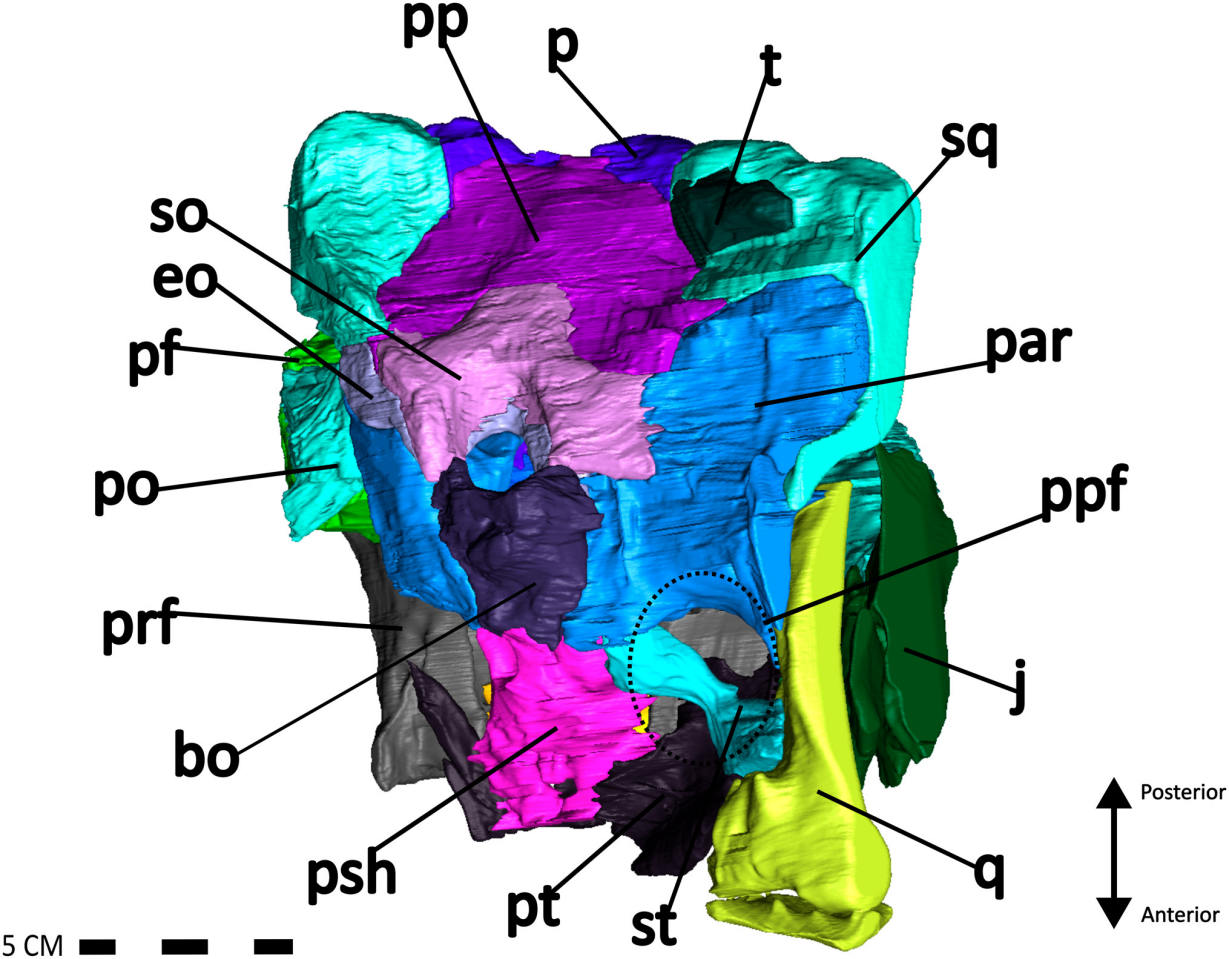
*Moschognathus whaitsi* (Capitanian, South Africa), juvenile specimen AM 4950, ventral view of posterior portion of skull. bo, basioccipital; eo, exoccipital; j, jugal; p, parietal; par, paroccipital; pf, postfrontal; po, postorbital; pp, postparietal; ppf, pterygoid‐paroccipital foramina; prf, prefrontal; psh, parasphenoid; pt, pterygoid; q, quadrate; so, supraoccipital; sq, squamosal; st, stapes; t, tabular. Scale bar equals 5 cm.

**FIGURE 5:**
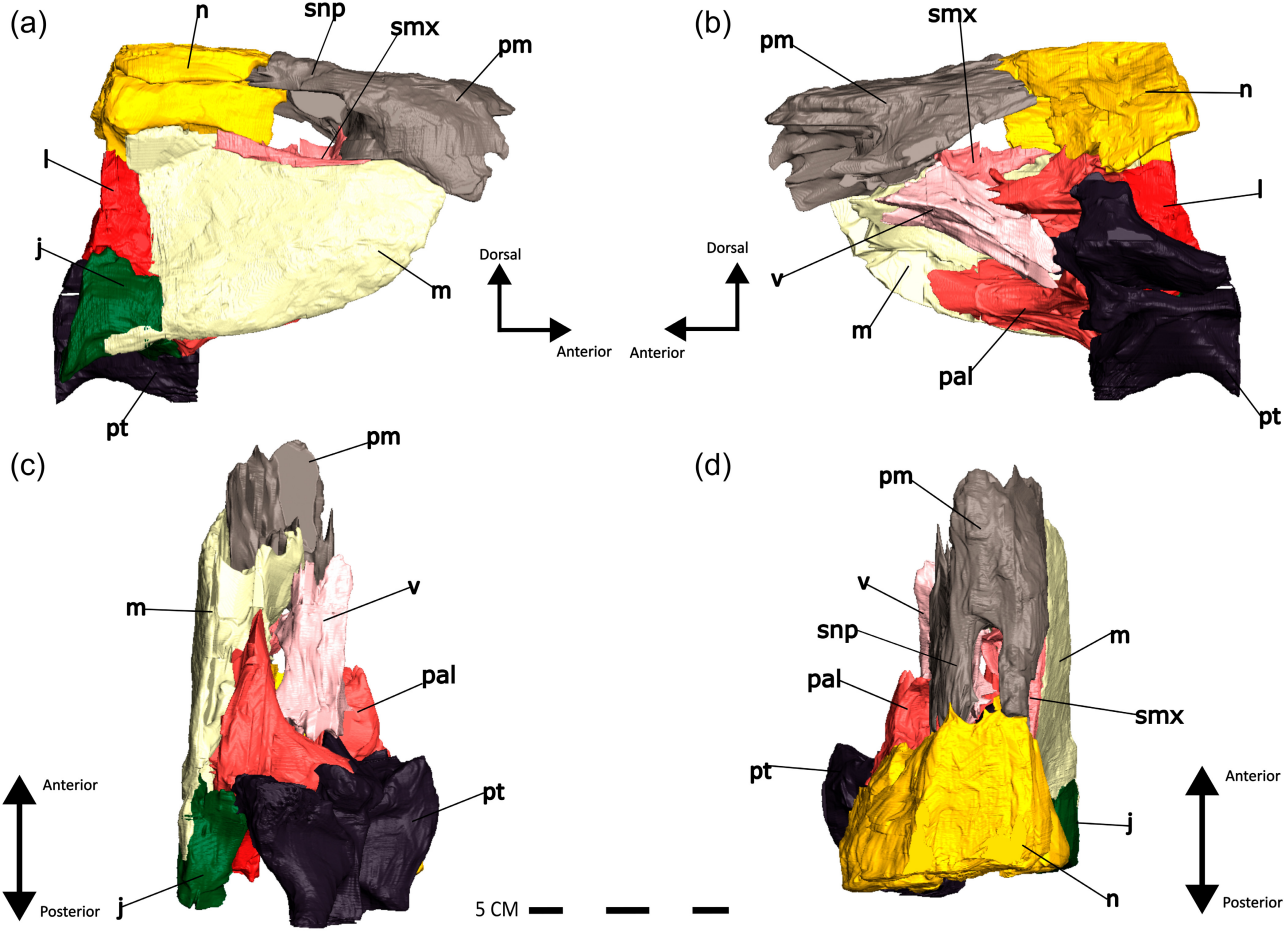
*Moschognathus whaitsi* (Capitanian, South Africa), juvenile specimen AM 4950, anterior portion of skull. (a) Right lateral view. (b) Right medial view. (c) ventral view. (d) Dorsal view. j, jugal; l, lacrimal; m, maxilla; n, nasal; pal, palatine; pm, premaxilla; pt, pterygoid; smx, septomaxilla; snp, supranarial process; v, vomer. Scale bar equals 5 cm.

When AM 4950 was being prepared, the right lateral margin of the frontal was damaged. The damage was refilled with plaster (Figure 1a). Specimen AM 4950 was also transversely broken in half (Figure 1); any taphonomic effects this has on the cranial bones will be mentioned in the descriptions. From a taphonomic standpoint, the right side of the skull is well preserved, whereas the left side has undergone heavy weathering, especially the left palate (Figures 1b, 3, 4 and 5b, c). Many bones on the left are missing, including the left maxilla, lacrimal, palatine, quadrate, jugal, stapes, tabular, dentary, angular, surangular, and prearticular. Hence, unless otherwise stated, the following description is based chiefly on observations from the right side of the skull. The posterior region of the skull is better preserved than the anterior region. Both the posterior process of the premaxilla and the anterior region of the vomer are missing. In CT images, the medial side of the mandible is highly fragmented, making bone identification difficult. Both the left and right articular and quadratojugal are absent due to the orientation of the specimen during scanning, which resulted in the posterior end of the mandible not being positioned within the beam. Both the left and right ectopterygoid are unidentifiable. The left pterygoid has been displaced and lies dorsal to the right pterygoid.

### 3.2 Premaxilla

The premaxilla is rectangular in lateral and dorsal view. It is represented by both premaxillae (Figures 1b and 5). Its posterior margin frames the anterior border of the external naris, and its main body forms most of the rostrum anterior to this opening. The supranarial process of the premaxilla is about half of the length of the main body of the bone, and extends slightly posterior to the external naris, of which it forms the entire dorsal margin (Figure 5a). It sutures with the maxilla anteroventrally, and septomaxilla posteroventrally, and the nasal posteriorly (Figure 5a, d). Furthermore, the septomaxilla's contact appears minimal in lateral view. None of the premaxilla's sutures are interdigitated. The anteroventral end contains five dental alveoli (Figure 5b, c). The dental alveoli and the teeth within them are inclined procumbently.

### 3.3 Septomaxilla

**FIGURE 6:**
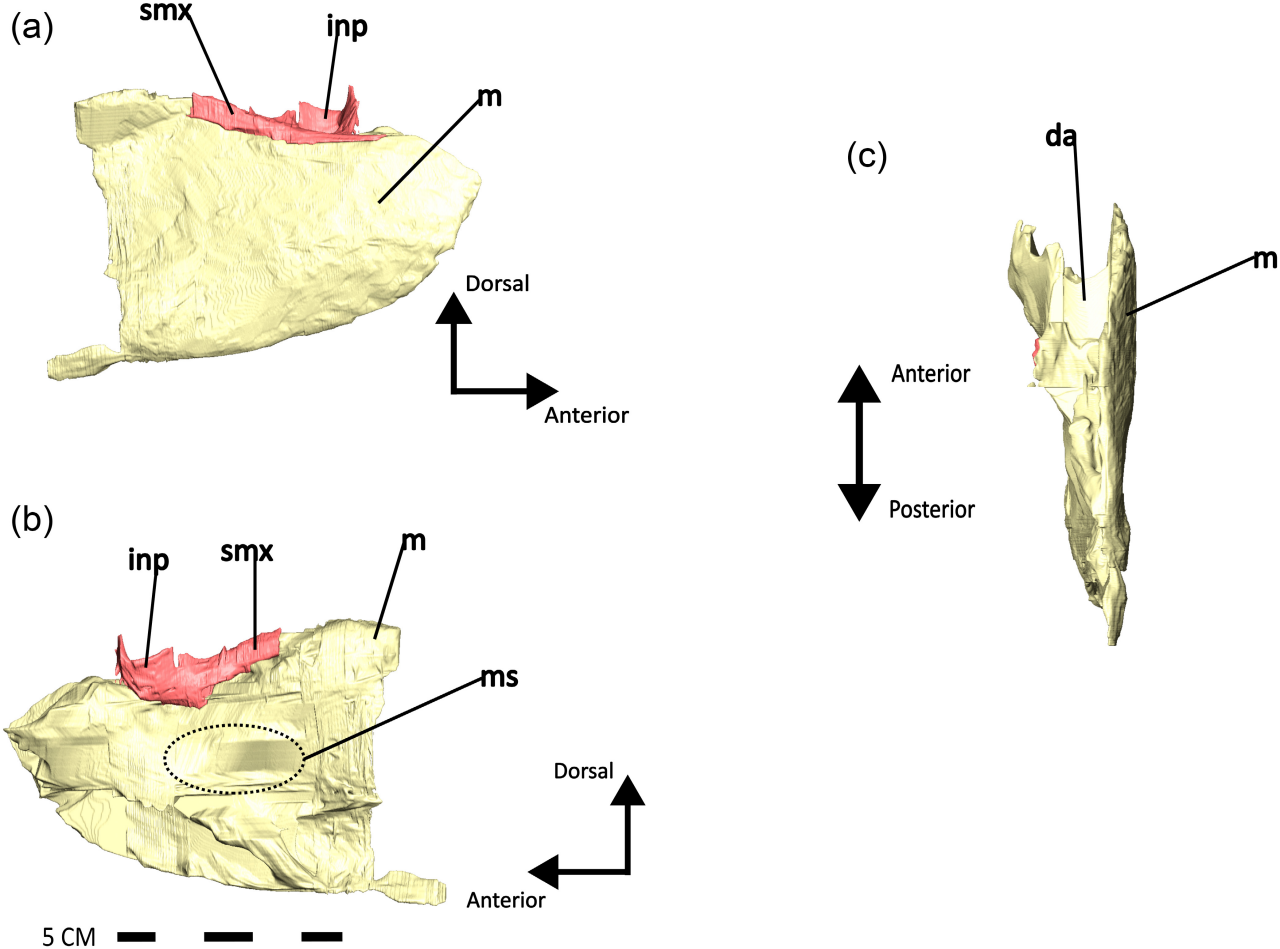
Right Maxilla and septomaxilla of AM 4950. (a) Lateral view. (b) Right medial view. (c) Ventral view. da, dental alveoli; inp, internarial process; m, maxilla; ms, maxillary sinus; smx, septomaxilla. Scale bar equals 5 cm.

The septomaxilla is thin and triangular in lateral view (Figures 1b and 5a). It forms the ventral margin of the naris. The septomaxilla sutures the premaxilla anterodorsally, the maxilla ventrally, and the nasal posterodorsally. None of the septomaxilla's sutures are interdigitated. In lateral view, the thickness of the septomaxilla increases from the anterior end to the posterior end. The internarial process is located on the anteromedial side of the septomaxilla. The internarial process is cup‐shaped and dorsal‐facing (Figure 6a, b).

### 3.4 Maxilla

In lateral view, the maxilla's alveolar margin curves anterodorsally such that the bone roughly has the shape of a right triangle (Figures 1b, 5 and 6). It sutures the premaxilla anterodorsally, the septomaxilla dorsally, the nasal posterodorsally, the lacrimal and palatine in its posteromedial side, and the jugal posteroventrally. None of the maxilla's sutures are interdigitated. In lateral view, the posterior lateral surface bears a pre‐orbital depression (possibly from postmortem damage). The medial surface has a depression that would have held the maxillary sinus (Figure 6b). A posteroventral process overlaps the jugal's anteroventral surface. The anteroventral edges contain nine dental alveoli. The mesial dental alveoli are as wide as the premaxilla and decrease in diameter distally (Figure 6c). As in the premaxilla, the ‘incisiform’ teeth of the maxilla project procumbently.

### 3.5 Nasal

**FIGURE 7:**
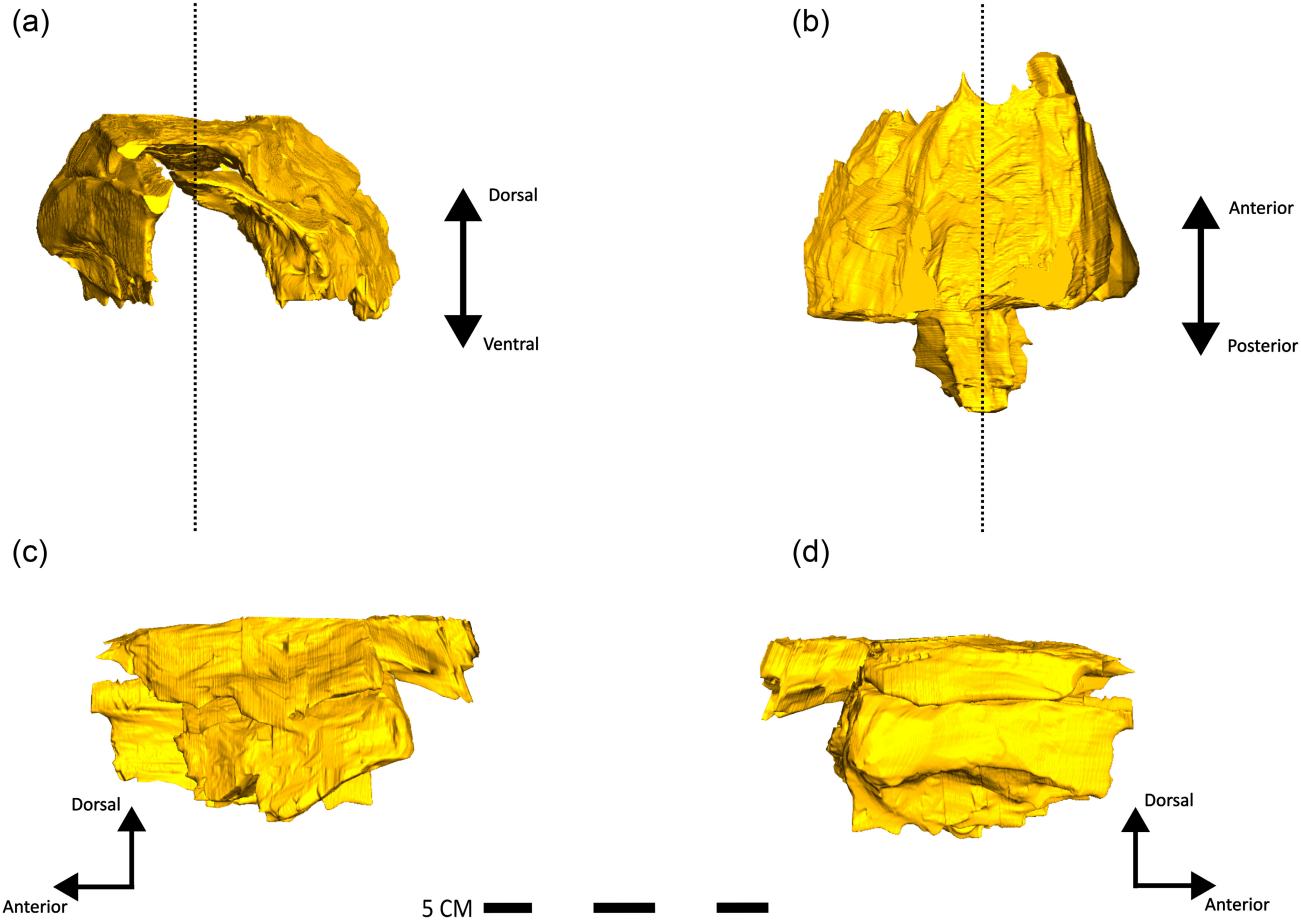
Nasal of AM 4950. (a) Anterior view. (b) Dorsal view. (c) Left lateral view. (d) Right lateral view. Scale bar equals 5 cm.

The nasal is a dorsally and laterally flat bone. It is represented by both nasals (Figures 1b, 3, 5 and 7). It should also be noted that AM 4950 was transversely broken in half along the nasal (Figure 1). The nasal sutures the premaxilla anteriorly, the septomaxilla anteroventrally, the maxilla ventrally, the prefrontal posterolaterally and posteriorly, and the frontal posteriorly. None of the nasal sutures are interdigitated. In anterior view, the nasals resemble an arch that forms the dorsal surface of the rostrum (Figures 5d and 7a). The anterolateral edge of the nasal forms the posterior rim of the naris. The posterior end of the nasal is a process situated between the prefrontals (Figures 3c and 7b).

### 3.6 Prefrontal

**FIGURE 8:**
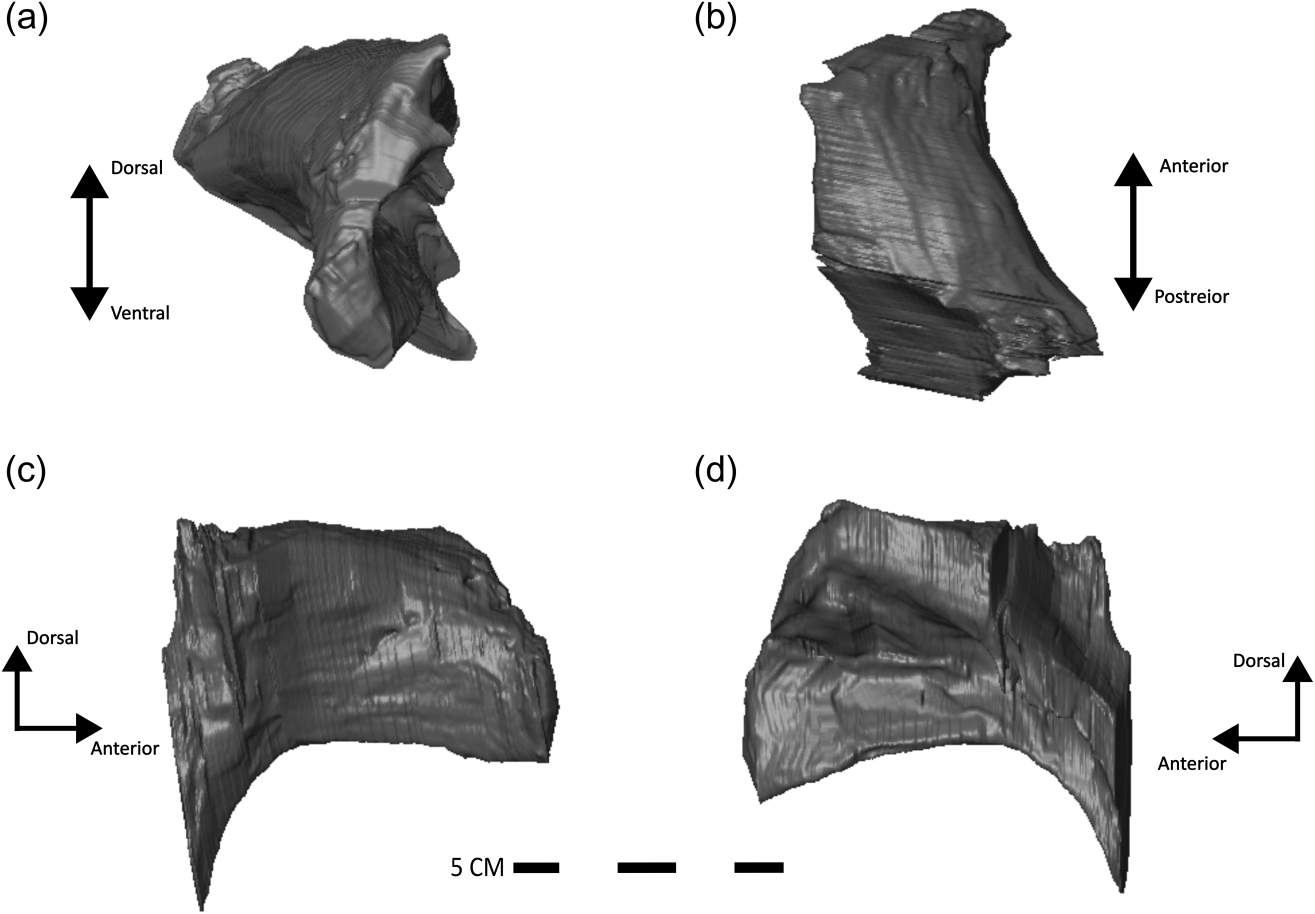
Right prefrontal AM 4950. (a) anterior view. (b) Dorsal view. (c) Lateral view. (d) Right medial view. Scale bar equals 5 cm.

The prefrontal is a large quadrangular bone that forms the dorsal rim of the orbit (Figures 1b, 3 and 8). In anterior view, the prefrontal forms an arch (Figure 8a). The prefrontal sutures the nasal anteriorly, the posterior process of the nasal medially, and the frontal posteriorly. None of the sutures are interdigitated. The prefrontal's suture with the lacrimal is lost since AM 4950 was transversely broken anterior to the prefrontal. The posterolateral end of the prefrontal and the anterolateral end of the postfrontal come into contact. This contact overlaps the portion of the frontal such that it does not contribute to the orbital margin externally.

### 3.7 Frontal

**FIGURE 9:**
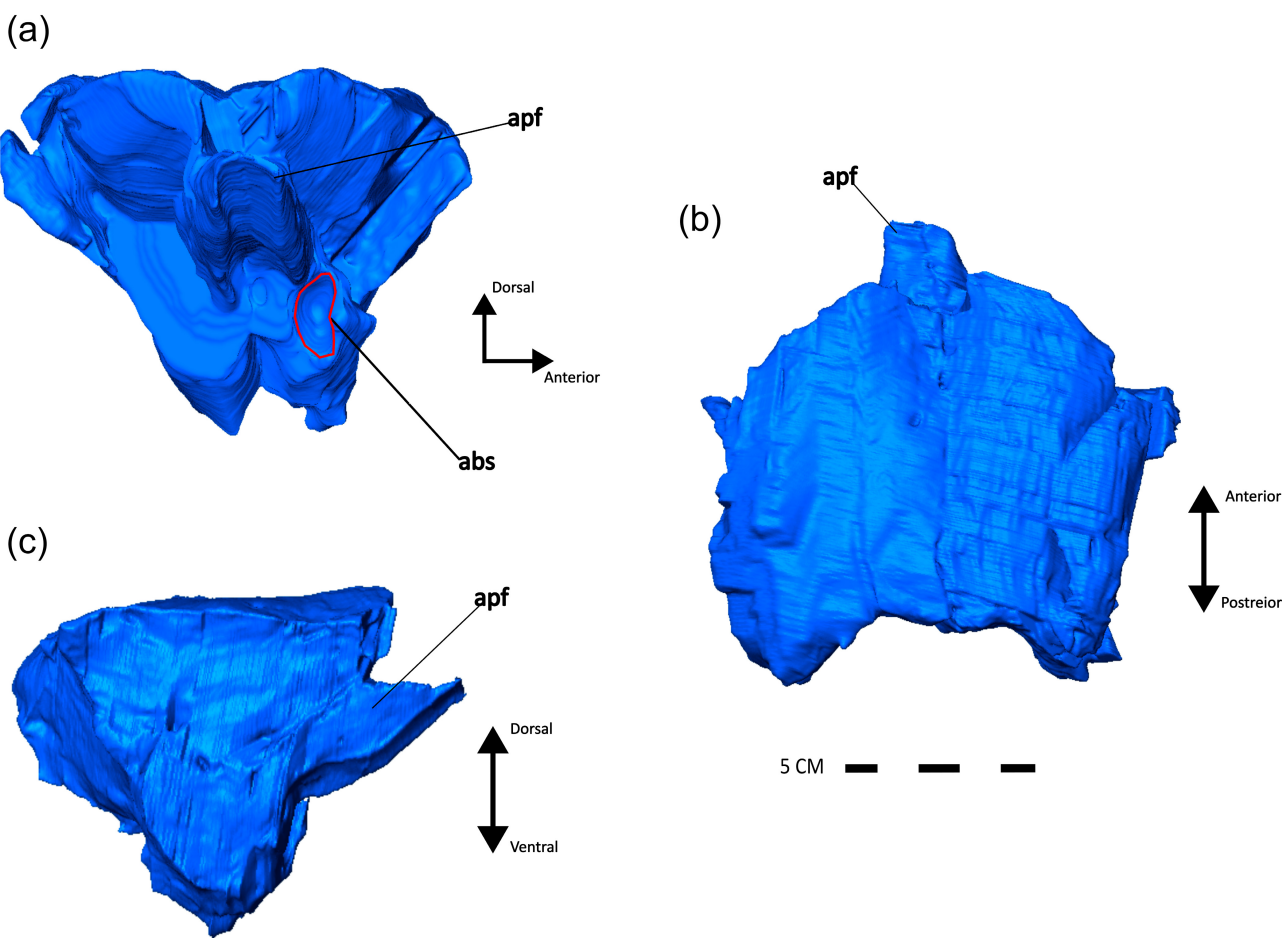
Frontal of AM 4950. (a) Anterior view. (b) Dorsal view. (c) Left lateral view of left frontal. abs, abscess; apf, anterior process of frontal. Scale bar equals 5 cm.

**FIGURE 10:**
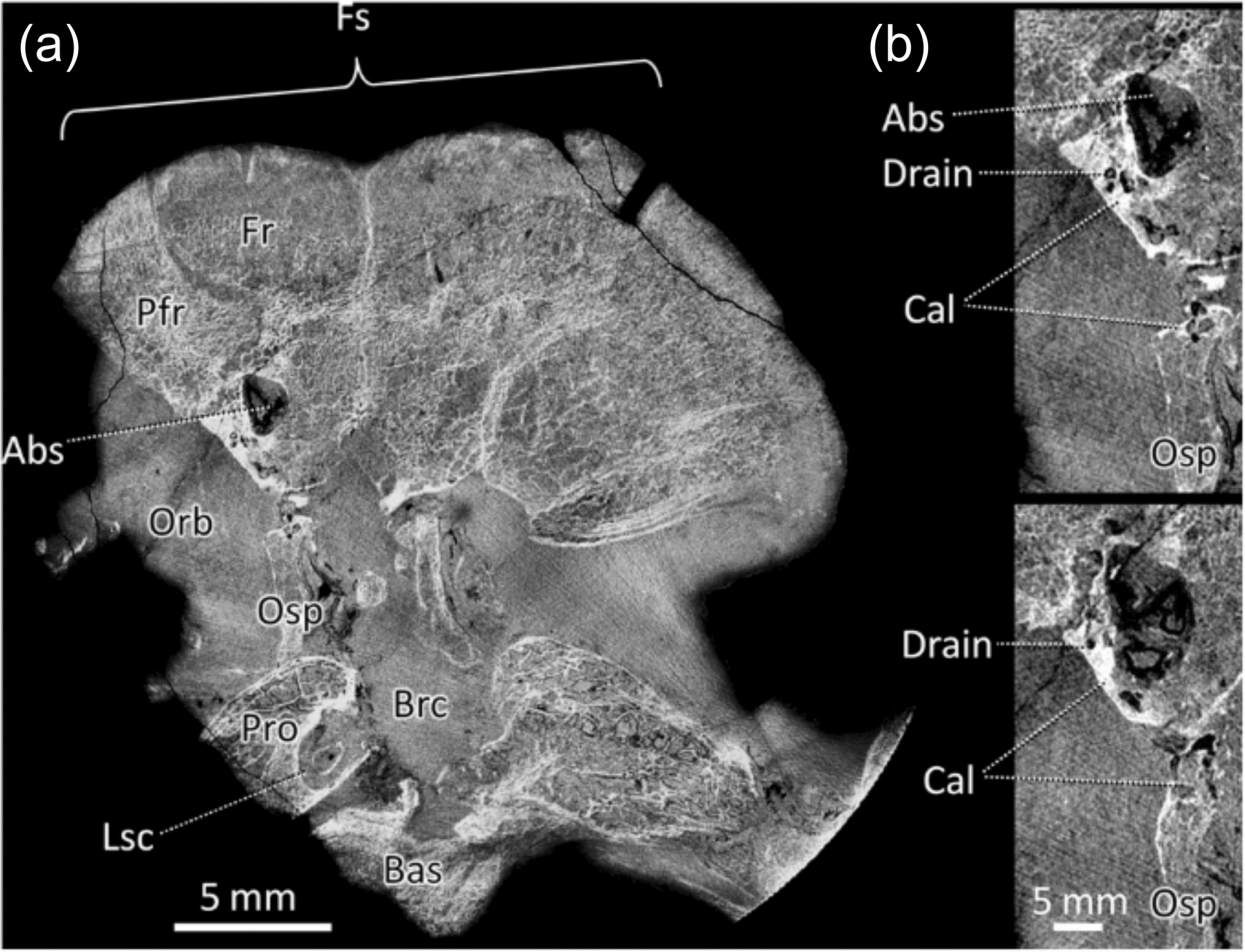
CT virtual cross‐section through the braincase of *Moschognathus whaitsi* (AM 4950). Abscess marked as Abs. (From Benoit et al., 2026). Scale bars equal 5 mm.

The frontal is the largest bone in the skull of AM 4950, forming most of the skull roof posterior to the orbit (Figure 1b). It is represented by both elements (Figures 1b, 3 and 9a, b). The frontal sutures the prefrontal anterolaterally and nasal anteromedially, the postfrontal laterally, and the parietal posteriorly. None of the sutures are interdigitated. The anterior face of the frontal bears an anterodorsally curved process (Figure 9). The ventral face of this process houses the frontal's anteroventral fossa. This fossa houses the orbitosphenoid's dorsal knob and would have also housed the olfactory bulbs (Figure 9a). A large abscess (~2 cm wide) is evident on the right anteroventral face of the frontal (Figures 9 and 10). Further details on the abscess are provided in the discussion section. The dorsal aspect of the frontal is pentagonal, and its surface is flat (Figure 9b). The frontal tapers in width ventrally up to its contribution to the dorsal margin of the endocranial cast. The frontal is excluded from the orbit by the contact between the prefrontal and postfrontal (Figures 1b and 3).

### 3.8 Postfrontal

**FIGURE 11:**
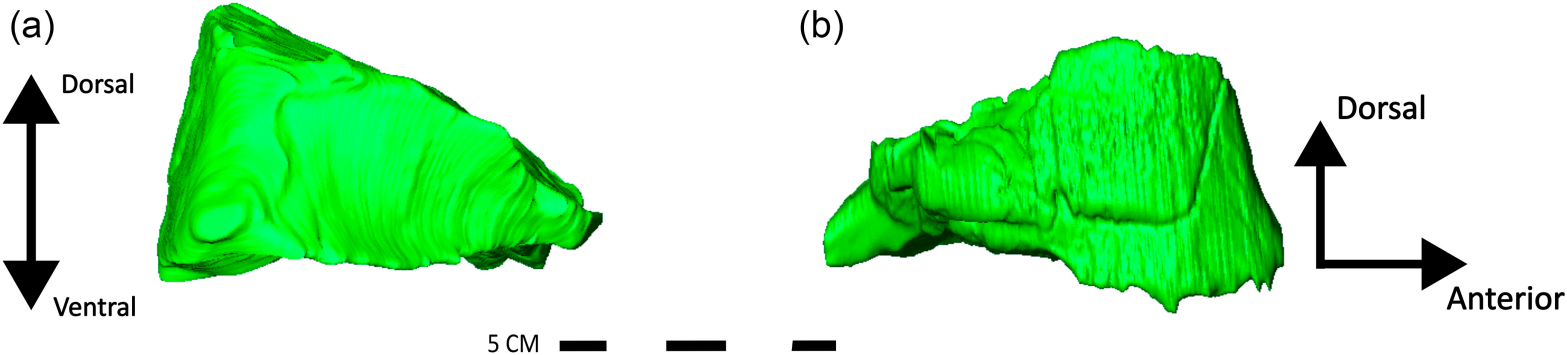
Right postfrontal. (a) Anterior view. (b) Lateral view. Scale bar equals 5 cm.

The postfrontal is a bulbous, horizontally tear‐shaped bone, tapering posteriorly and medially (Figure 11). It sutures the frontal dorsomedially, the postorbital ventrally, and the parietal posteriorly. None of the sutures are interdigitated. The anterior end of the postfrontal forms the posterior margin of the orbit (Figures 1b and 3). It also forms the dorsal half of the postorbital bar. The anterolateral end of the postfrontal and the posterolateral end of the prefrontal come into contact, excluding the frontal from the orbital margin. The posterior ventral region of the postfrontal forms the dorsal margin of the temporal fenestra (Figures 1b and 3).

### 3.9 Lacrimal

**FIGURE 12:**
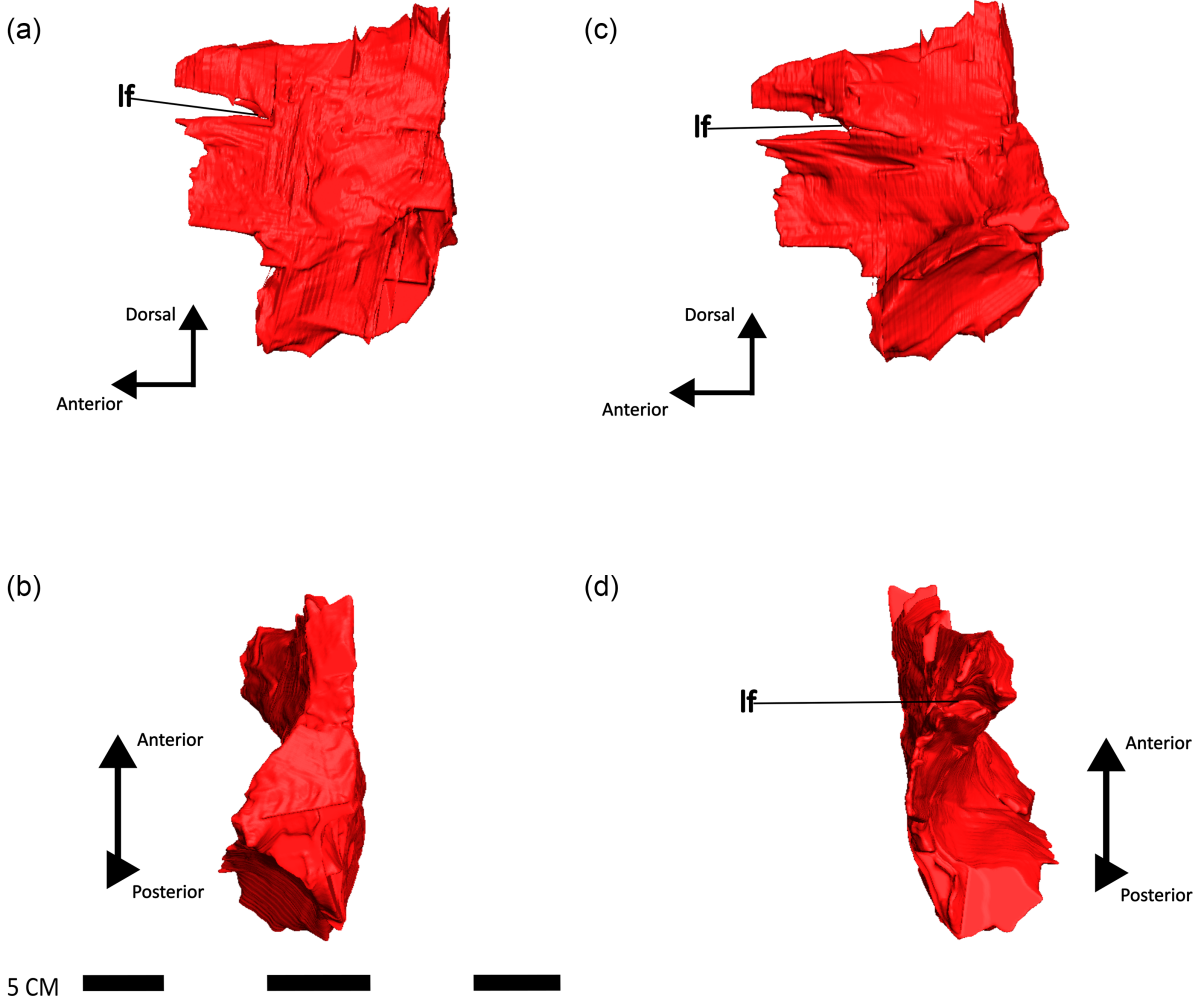
Right lacrimal of AM 4950. (a) Lateral view. (b) Posterior view. (c) Medial view. (d) Anterior view. lf, lacrimal foramen. Scale bar equals 5 cm.

In lateral view, the lacrimal is quadrangular and forms the anterior margin of the orbit (Figures 1b, 5, and 12). The lacrimal sutures the maxilla anteriorly, the nasal dorsally, and the jugal posteroventrally. None of the sutures are interdigitated. The contact between the lacrimal and prefrontal is not present as this region of the posterior portion of AM 4950 was not exposed to the beam during scanning (Figures 1b and 3). In lateral view, the lacrimal is overlapped by the maxilla anteriorly. A shallow lacrimal fossa excavates its lateral surface. Its anterior margin is notched by a single lacrimal foramen. Posteriorly, the lacrimal forms the anterior margin of the orbit. In medial view, the ventral most aspect of the lacrimal is slightly concave (Figure 12c, d).

### 3.10 Postorbital

**FIGURE 13:**
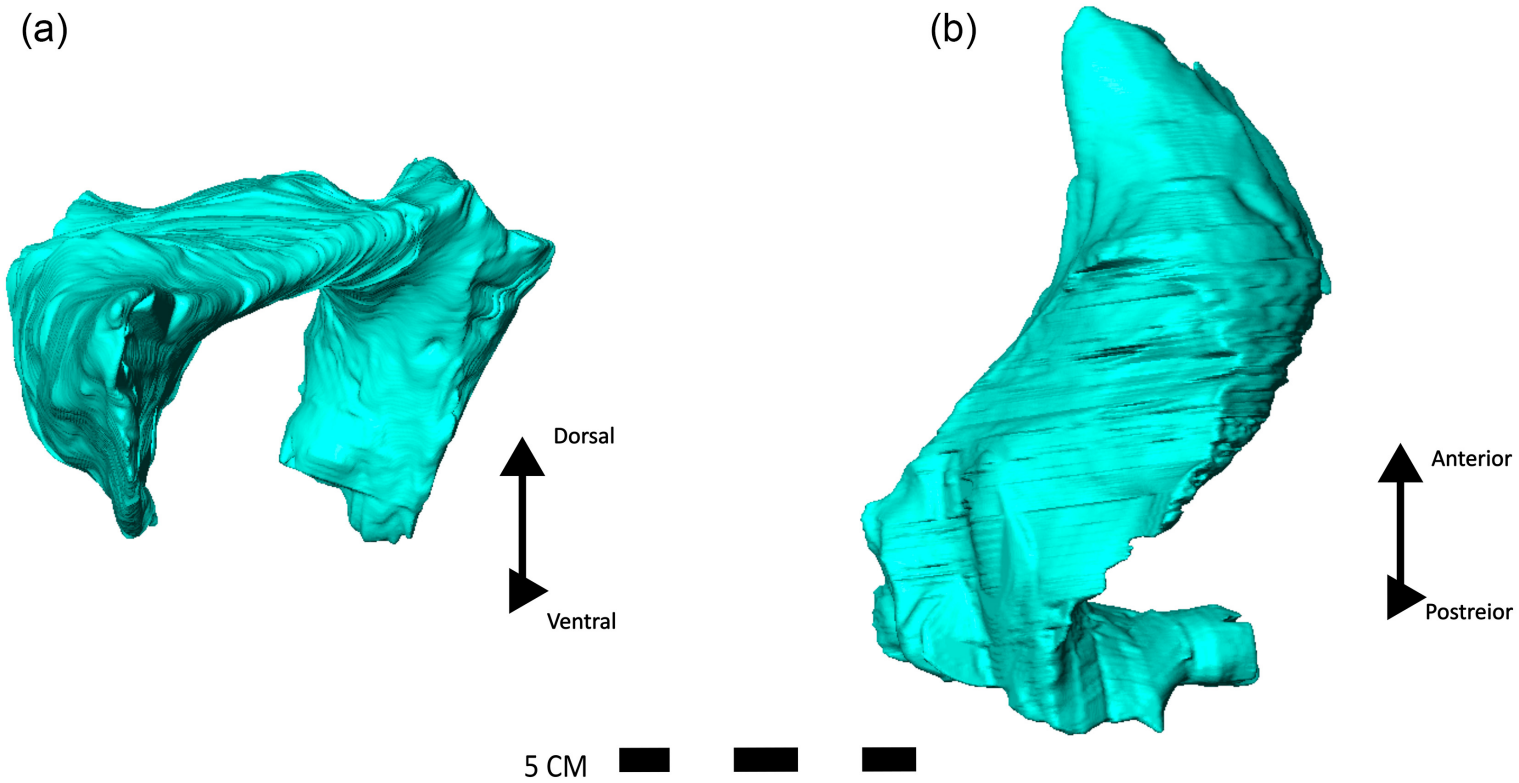
Right postorbitals of AM 4950. (a) Anterior view. (b) Dorsal view. Scale bar equals 5 cm.

The postorbital is laterally elongated and spans most of the length of the temporal region (Figures 3 and 13). It curves anteroventrally in a short tapering process. It sutures the jugal anteroventrally, the postfrontal dorsally, the squamosal posterolaterally, the parietal posteriorly, and the exoccipital posteroventrally. None of the sutures are interdigitated. The postorbital can be divided into an anterior and posterior half. Its anterior half forms the ventral half of the postorbital bar and the posteroventral margin of the orbit (Figure 3). In lateral view, the posterolateral half curves concavely to form the anterior and dorsal margins of the temporal fenestra. The posteriormost end curves ventrolaterally (Figure 13).

### 3.11 Jugal

**FIGURE 14:**
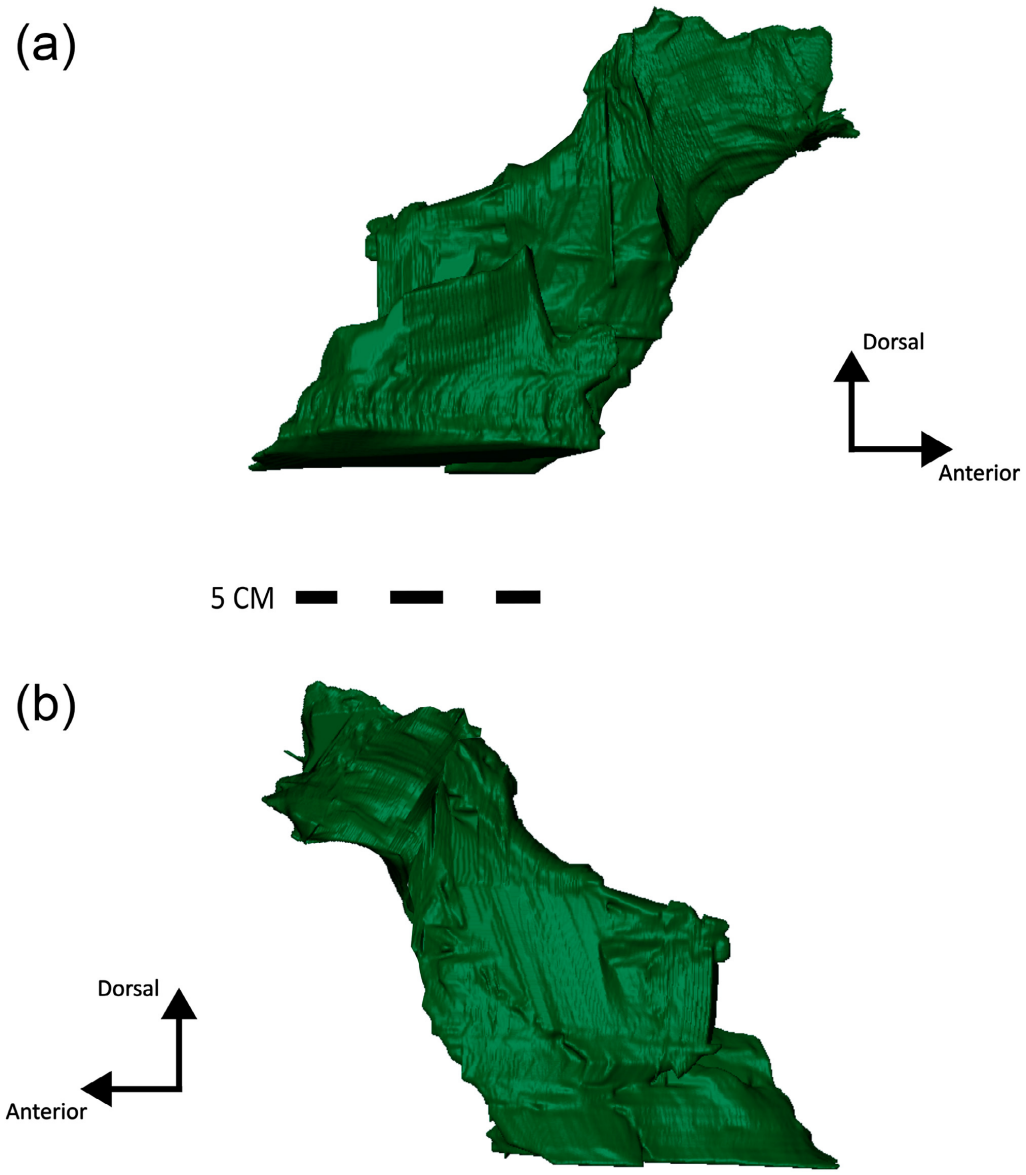
Right Jugal of AM 4950. (a) Lateral view. (b) Medial view. Scale bar equals 5 cm.

Due to the position of the break in AM 4950, the first quarter of the jugal is separated from the rest of the bone. The posteroventral surface appears artificially flat due to how AM 4950 was positioned in the beam during the CT scanning process (Figure 14). The jugal is a laterally elongated and transversally thin bone that forms the ventral margin of the skull externally, spanning from the level of the anterior orbital margin to nearly the posterior margin of the skull (Figure 14). It sutures the maxilla anteriorly, the lacrimal anterodorsally, and the postorbital posterodorsally. None of the sutures are interdigitated. In lateral view, the jugal has been broken in half, possibly from compression. The anterior half's lateral surface is overlapped by the posterior half's medial surface. The jugal forms the anteroventral margin of the orbit. In lateral view, the anterior end curves upward along the orbital rim (Figure 3). The anteroventral face is concave, possibly to accommodate the surangular when the jaw is closed (Figure 1b). The posterior end forms the anteroventral margin of the temporal fenestra.

### 3.12 Squamosal

**FIGURE 15:**
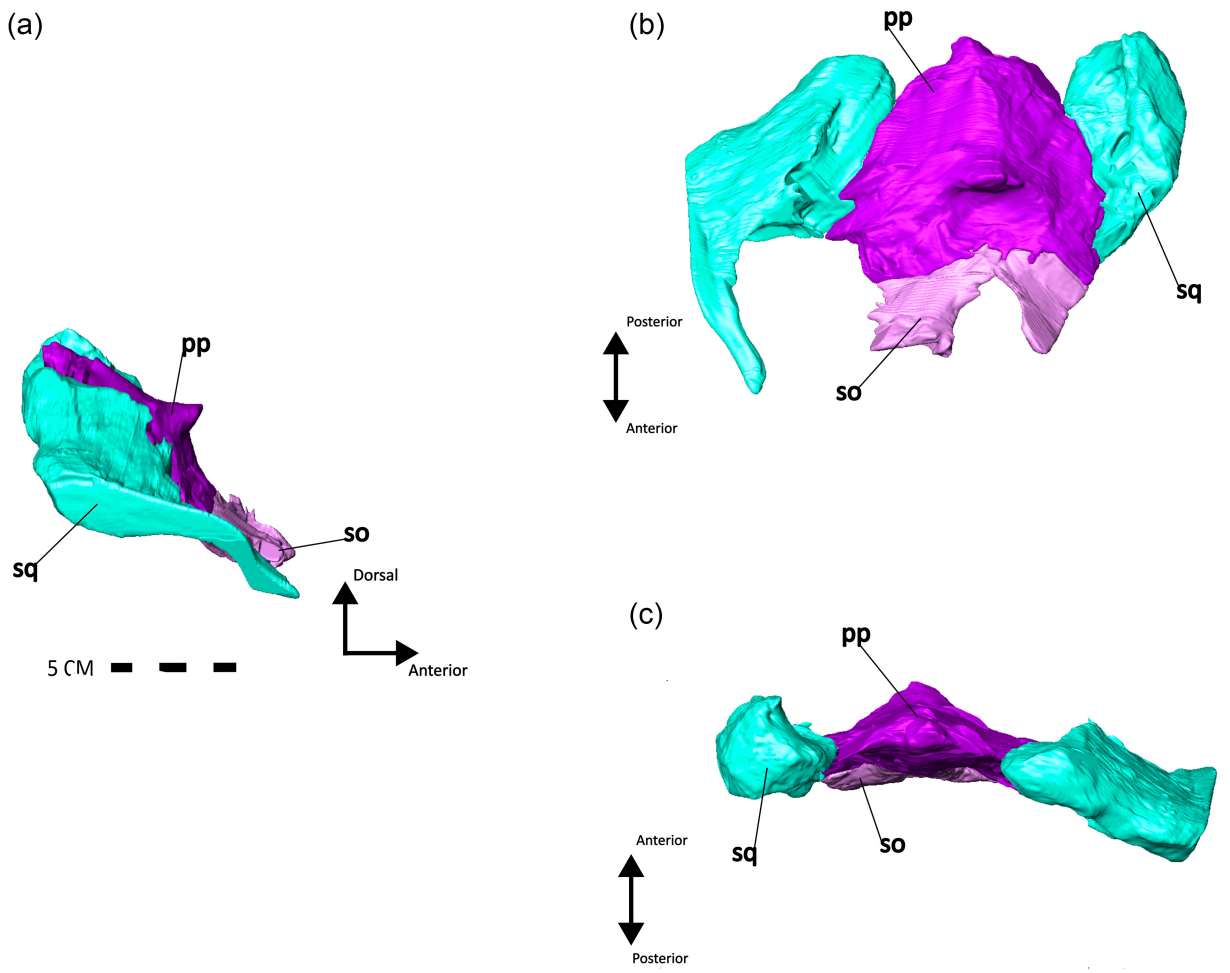
Postparietal, supraoccipital, and squamosal of AM 4950. (a) Right lateral view. (b) Anterior view. (c) Dorsal view. pp, postparietal; sq, squamosal; so, supraoccipital. Scale bar equals 5 cm.

The squamosal's lateral surface appears flat, due to how AM 4950 was positioned during the CT‐scanning process. The posterior face is exposed, due to the weathering of the tabular. The squamosal is an anteroposteriorly narrow, mediolaterally wide bone. It is located at the posterolateral end of the skull (Figures 4 and 15). The main body of the bone is transversely wide, forming most of the width of the posterior skull. It sutures with the opisthotic and exoccipital anteriorly, the parietal dorsally, the postorbital medially, and the tabular posteriorly. None of the sutures are interdigitated. The anterior surface of the main body is flat. The squamosal also bears a thin and tapering anteroventral process that extends to the ventral margin of the skull externally, forming an extensive suture with the paroccipital medially. This process forms the posterior margin of the temporal fenestra. The anterior end forms the posterior margin of the temporal fenestra.

### 3.13 Parietal

**FIGURE 16:**
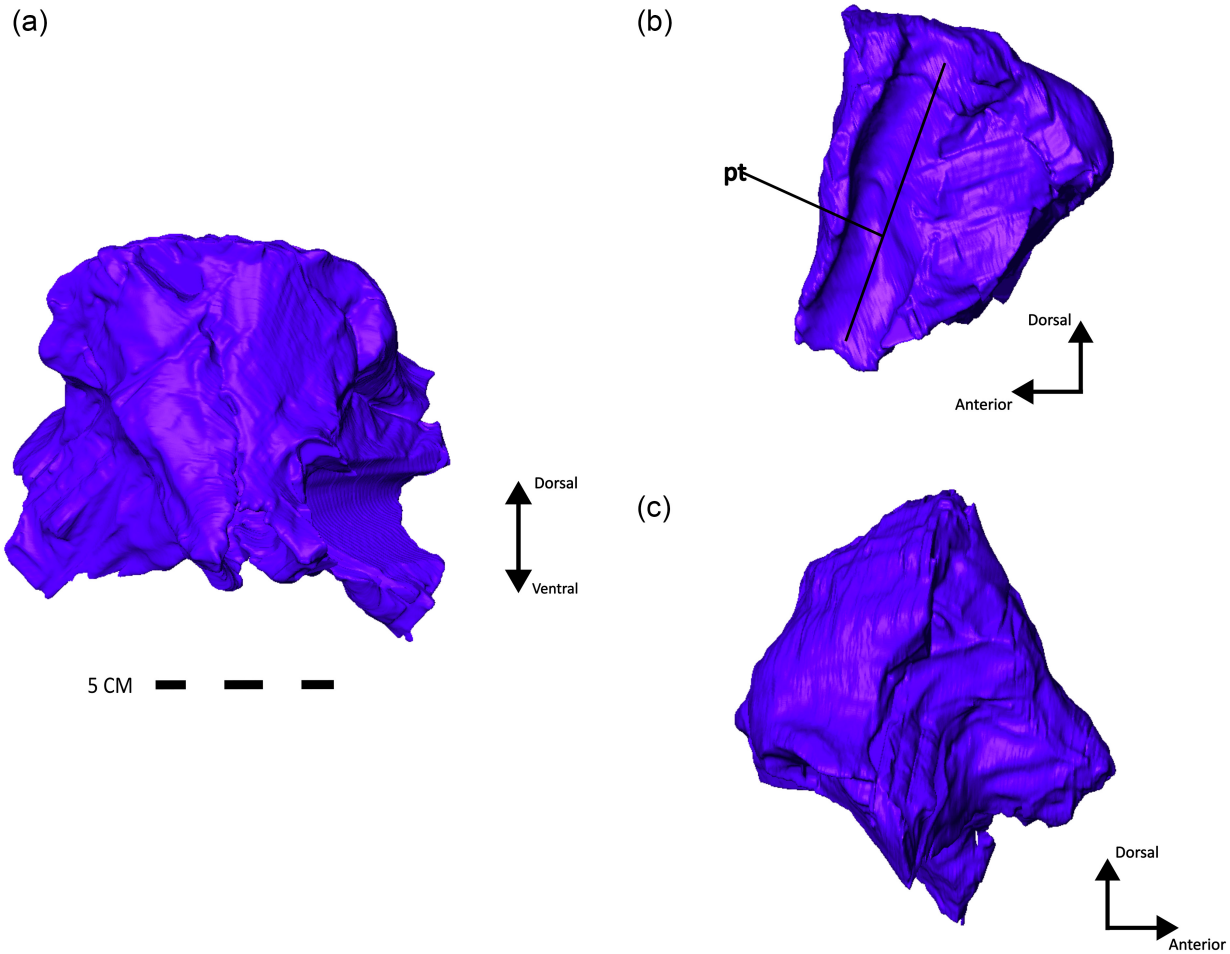
Parietal of AM 4950. (a) Anterior view. (b) Right interior view. (c) Left lateral view. pt, pineal tube. Scale bar equals 5 cm.

The parietal is the second largest bone in the skull roof and forms the posteromedian margin of the skull (Figures 1b, 3, and 16). The parietal is represented by both bones (Figures 1b, 3c, and 16a). It sutures the frontal anteriorly, the postfrontal and the postorbital anterolaterally, the exoccipital ventrally, the squamosal laterally, and the postparietal posteroventrally. There are no interdigitated sutures. In anterior view, the parietal is conical (Figure 16a). The dorsal surface of the bone is curved. Two posterior processes extend ventrolaterally and suture with the squamosal. In lateral view, the pineal tube is situated in the anterior half of the parietal (Figure 16c). The dorsal surface is curved. The ventral end of the pineal tube opens into the endocranial space.

### 3.14 Postparietal

The postparietal is an unpaired, flat, oval‐shaped bone. It forms most of the dorsal surface of the occiput (Figures 4 and 15). It sutures the parietal anterodorsally and the squamosal and tabular laterally. None of the sutures are interdigitated. The anterior surface is slightly convex (Figure 15c) and sutures the opisthotic anterolaterally. The anterior face has a small socket with a pronounced dorsal lip for articulating with the exoccipital (Figure 16b). The ventral extent of the bone is overlapped by the supraoccipital. The posterior face of the postparietal is flat, with a faint medial ridge that extends from the supraoccipital to the most dorsal point.

### 3.15 Tabular

The tabular of AM 4950 is located in the right posterodorsal corner of the occiput (Figure 4). It is a poorly preserved, irregular knob shape. What remains of the tabular shares an anterior suture with the squamosal and a lateral suture with the postparietal. None of the sutures are interdigitated.

### 3.16 Supraoccipital

The supraoccipital is an anteroposteriorly narrow bone that forms the dorsal margin of the foramen magnum (Figures 4 and 15). It sutures the postparietal anterodorsally, the exoccipital anteriorly, the basioccipital and prootic (labeled in Figure 4 as paroccipital) anteroventrally, and the opisthotic laterally. None of the sutures are interdigitated. It consists of a thin lamina with slightly expanded lateral flanks and wraps around the ventral margin of the postparietal. Dorsally, it extends as a pointed process that overlaps with the postparietal. Its ventral end forms the dorsal margin of the foramen magnum (Figure 4).

### 3.17 Basioccipital

The basioccipital is a small, irregular bone (Figure 4), located at the posteroventral end of AM 4950. It forms the ventral‐most end of the braincase. It also forms the ventral margin of the foramen magnum. It sutures the parasphenoid anteriorly, the prootic laterally, and the supraoccipital posteriorly. The basioccipital bears tall basal tubera that extend anteriorly along the anteromedial margins of the paroccipital to reach the posterior margin of the parasphenoid. Posteroventrally, the basioccipital bears a short neck terminating in the occipital condyle.

### 3.18 Quadrate

**FIGURE 17:**
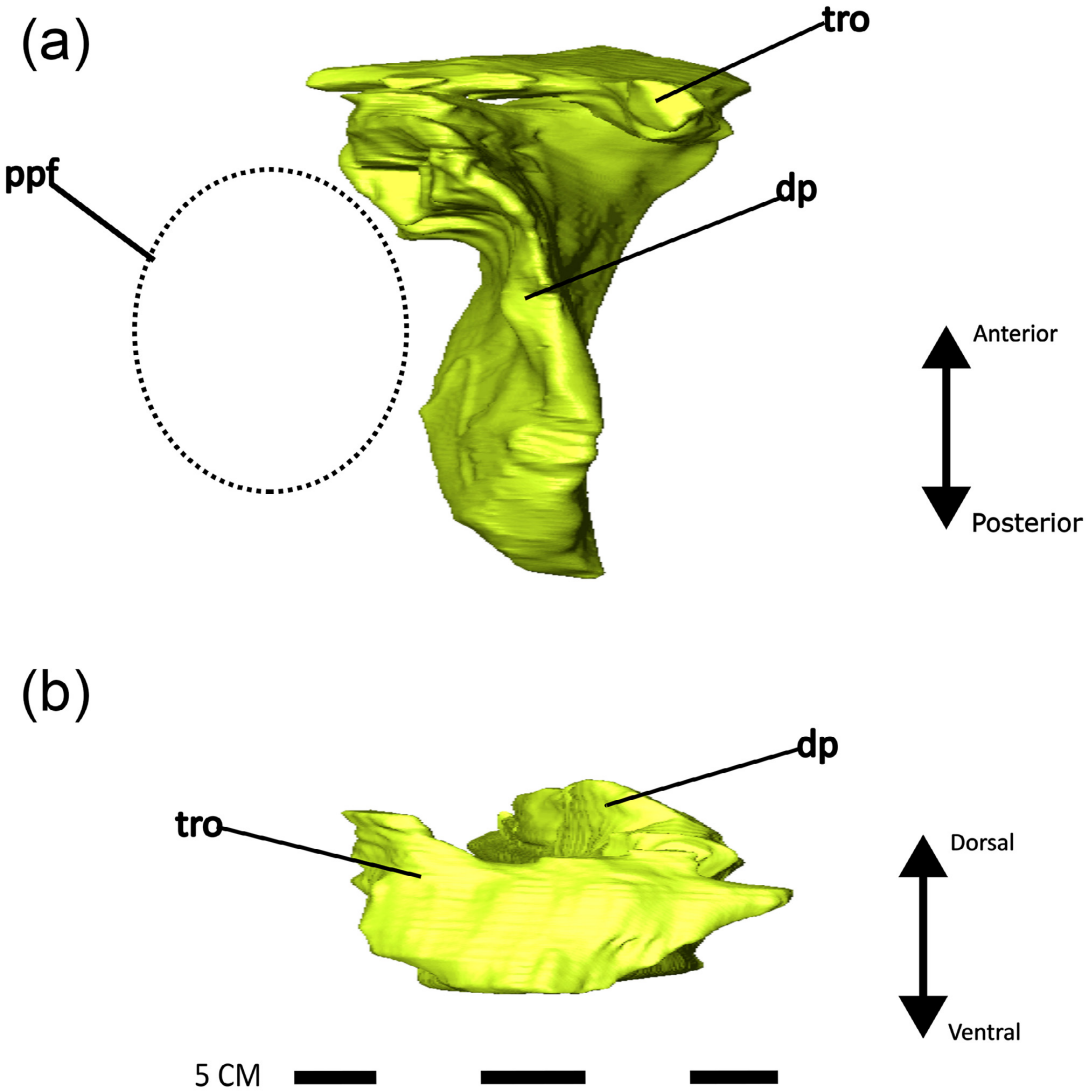
Right quadrate of AM 4950. (a) Dorsal view. (b) Anterior view. dp, dorsal plate; ppf, pterygoid‐paroccipital foramina; tro, trochlea. Scale bar equals 5 cm.

The quadrate is elongated and strongly twisted (Figure 17). In dorsal view, the quadrate is hourglass‐shaped (Figure 17a). The anterior face is broader than the posterior end. It sutures with the stapes anteromedially and the prootic posteromedially. None of the sutures are interdigitated. It abuts the ventral end of the quadrate ramus of the pterygoid (Figure 4). The trochlea is an anteroposteriorly narrow plate that articulates with the quadrate's anterior face (Figure 17). The anterior face is broader than the posterior end. The medial surface of the quadrate forms the lateral margins of the pterygoid‐paroccipital foramina (Brink, 1958). The dorsal plate is strongly twisted. The ventral surface of the quadrate appears flat due to how AM 4950 was positioned during the CT scanning process. The posterior end comes to a point in both ventral and dorsal view.

### 3.19 Vomer

**FIGURE 18:**
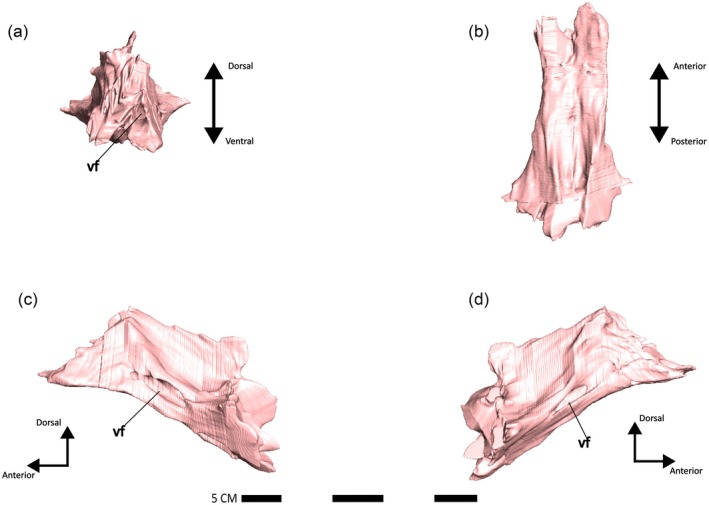
Vomer. (a) Anterior view. (b) Ventral view. (c) Left lateral view. (d) Right lateral view. vf, vomer foramina. Scale bar equals 5 cm.

The vomer is rectangular and located in the anteromedial region of the skull (Figure 18). The vomers are partially fused. The sutures with the surrounding bones have been lost due to weathering. It abuts the palatine posterolaterally and the pterygoid posteriorly. The anterior end is missing. In anterior view, one of the left vomer foramina is visible. The posterolateral ends have wing‐shaped processes (Figure 18a, b). Both the lateral surfaces have been weathered enough so that both the vomer foramina are visible. The dorsal surface has a medial keel that runs throughout the entire length of the vomer (Figure 18b). The ventral surface has a medial groove that runs the entire length of the vomer and is the only visible suture between the two vomers. The posterior surface is heavily weathered, precluding a detailed description of its contact with the palatine and pterygoid.

### 3.20 Palatine

**FIGURE 19:**
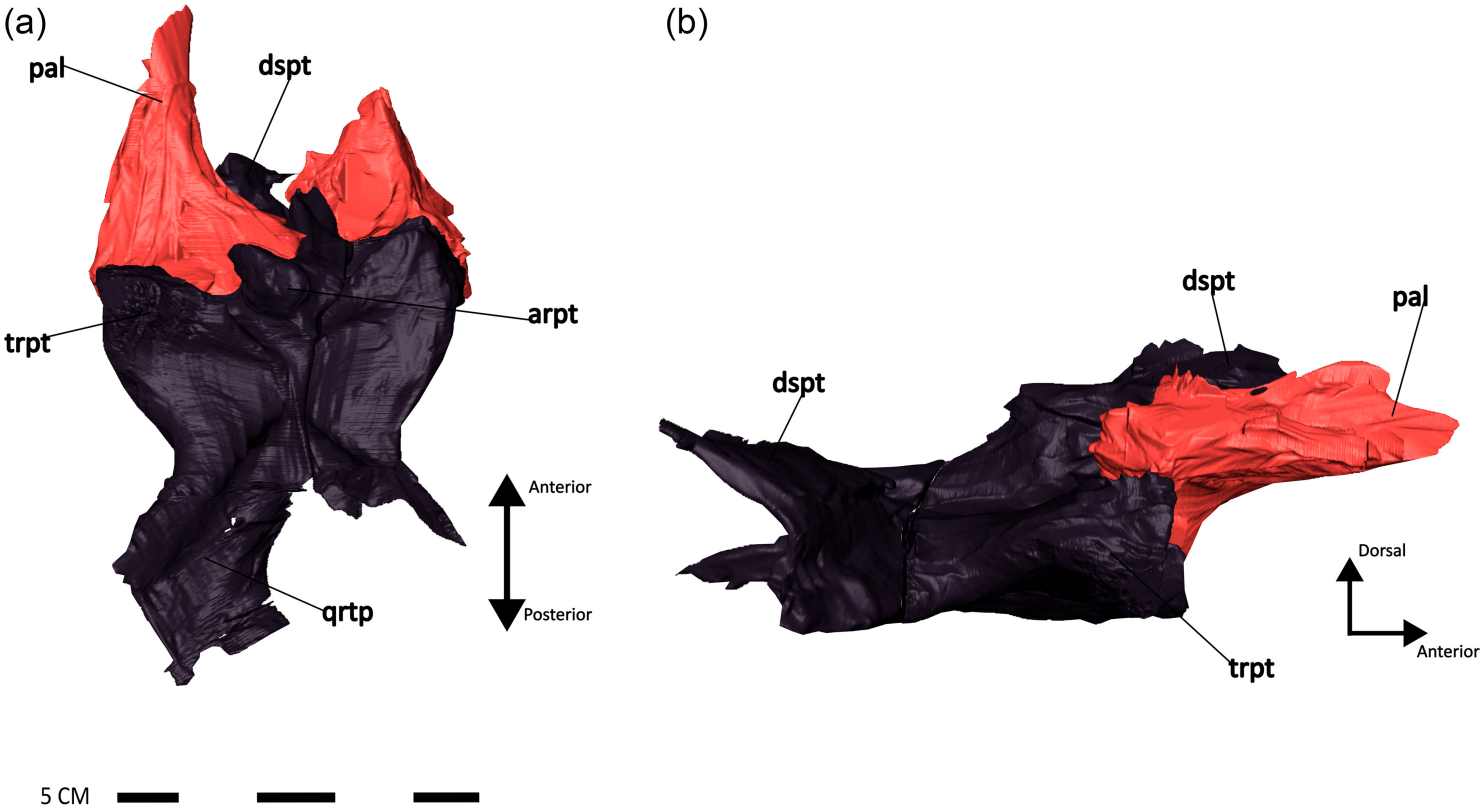
Palatine and Pterygoid of AM 4950. (a) Ventral view. (b) Right lateral view. arpt, anterior ramus of the pterygoid; dspt, dorsal septum of the pterygoid; pal, palatine; pt, pterygoid; qrpt, quadrate ramus of the pterygoid; trpt, transverse ramus of the pterygoid. Scale bar equals 5 cm.

The palatine is triangular (Figure 19) and forms the palate posterior to the vomer (Figure 5c). The palatine is thin with a flat dorsal and ventral surface. It has no palatal teeth or palatal bosses. It sutures the maxilla laterally. It abuts the pterygoid posteriorly. None of the sutures are interdigitated. The anterior end is pointed. The lateral edge is flat. The medial side is concavely curved, forming the lateral rim of the palatal foramen. Both the dorsal and ventral surfaces are flat. The posterior face is flat, where it abuts the pterygoid.

### 3.21 Pterygoid

The pterygoid is a tripartite bone, forming the posterior part of the palate (Figures 5 and 19). Both pterygoids appear to have extensive midline contact, forming an X‐shaped structure in ventral view (Figure 19a). However, the left pterygoid has been pushed above the right pterygoid. It is divided between the anterior, transverse, quadrate rami, and dorsal septum. It does not suture with any of the surrounding bones. The pterygoid abuts the palatal anteriorly and the parasphenoid posteriorly. The anterior ramus is short, reduced, spherical and abuts the palatine anteriorly. In ventral view, the anterior ramus is obscured by the posterior end of the palatine. The transverse ramus is mediolaterally narrow (a feature common in tapinocephalids; Boonstra, 1969) and bears a strong transverse ridge. The dorsal septum is heavily weathered, such that only the base is preserved. In ventral view, the quadrate ramus of the pterygoid expands in a posterolateral direction. The pterygoid's posterior end forms the anterior margin of the pterygoid‐paroccipital foramina (Figure 4).

### 3.22 Stapes

The stapes is small and wing‐shaped (Figure 4). It sits within the pterygoid‐paroccipital foramina. It is situated in the foramina at a posteromedial to anterolateral angle. It sutures the quadrate anterolaterally, the parasphenoid posterolaterally, and the prootic posteriorly. None of the sutures are interdigitated. The footplate forms the anterior end. It is broad and flat along the medial surface of the quadrate. The medial region of the stapes is the narrowest. The ventral surface is concave. The bulbous posterior end is situated in the fenestra ovalis.

### 3.23 Parasphenoid

**FIGURE 20:**
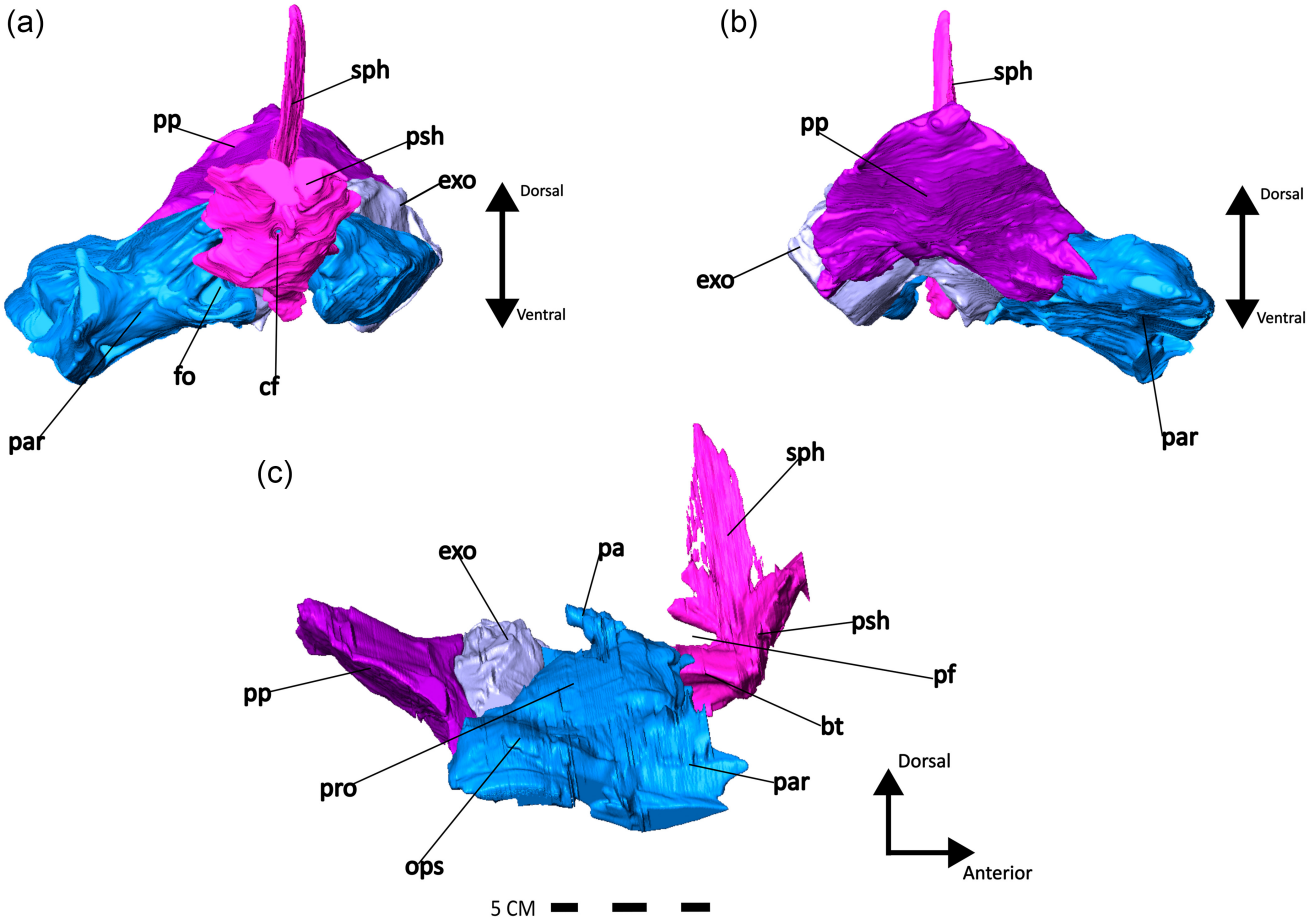
Exoccipital, paroccipital, postparietal, and parasphenoid/sphenethmoid of AM 4950. (a) Anterior view. (b) Posterior view. (c) Lateral view. bt, basisphenoid tubera; cf, carotid foramina; exo, exoccipital; fo, fenestra ovalis; ops, opisthotic; pa, pila antotica; par, paroccipital; pf, pituitary fossa; pp, postparietal; pro, prootic; psh, parasphenoid; sph, sphenethmoid. Scale bar equals 5 cm.

The parasphenoid is elongated and dorsally curving (Figures 4 and 20). It sutures the basioccipital posteriorly and the stapes and prootic posterolaterally. None of the sutures are interdigitated. The anterior tip is missing. In anterior view, two short processes project anteriorly from the anteriormost end. In anterior view, the two carotid foramina are small and located in the mid‐anteroventral area (Figure 20a). In lateral view, the anterior end curves dorsally (Figure 20c). The parasphenoid's dorsal surface holds the sphenethmoid. The sphenethmoid is a transversely thin, tall, fan‐shaped process. The sphenethmoid process bears a slightly thicker posterodorsal projecting processes. The posteroventral surface bears the basisphenoid tubera.

### 3.24 Paroccipital

The paroccipital is a combination of the prootic and opisthotic that forms the posteroventral plate of the skull (Brink, 1958). Although the paroccipital is often described as one bone in other descriptions, here both bones are described individually.

### 3.25 Prootic

The prootic is quadrangular and located on the posteroventral side of the skull (Figures 4 and 20). It sutures the parasphenoid anteriorly, the quadrate laterally, and the opisthotic and exoccipital posteriorly. None of the sutures are interdigitated. The sutures between prootic and opisthotic are indistinguishable. The anterior face houses the fenestra ovalis (Figure 20a). The anterolateral side forms the posterior margin of the pterygoid‐paroccipital foramina (Figure 4). The prootic's lateral surface appears flat due to how AM 4950 was positioned during the CT‐scanning process. The dorsal face houses the pila antotica. The pila antotica is a posteriorly projecting process (Figure 20c). The ventral surface is flat.

### 3.26 Opisthotic

The opisthotic is a dorsoventrally expanded bone (Figures 4 and 20) that sutures the prootic anteriorly, the squamosal laterally and posteriorly, and the exoccipital and postparietal posteromedially. None of the sutures are interdigitated. The sutures between prootic and opisthotic are indistinguishable. The opisthotic's lateral surface is flat due to how AM 4950 was positioned during the CT‐scanning process. In ventral view, the opisthotic is circular (Figure 4). The ventral surface is slightly concave.

### 3.27 Exoccipital

The exoccipital is quadrangular and forms the posterior end of the braincase (Figure 20c). It sutures the prootic anteriorly, the opisthotic laterally, the parietal and postorbital dorsally, the postparietal and squamosal posteriorly, and the supraoccipital posteroventrally. None of the sutures are interdigitated. The anterior face is at an angled slope. The dorsal surface is irregular. The ventral end is more angular. The anteroventral end is slightly bifurcated by the opening of the foramen magnum (Figure 20b).

### 3.28 Orbitosphenoid

**FIGURE 21:**
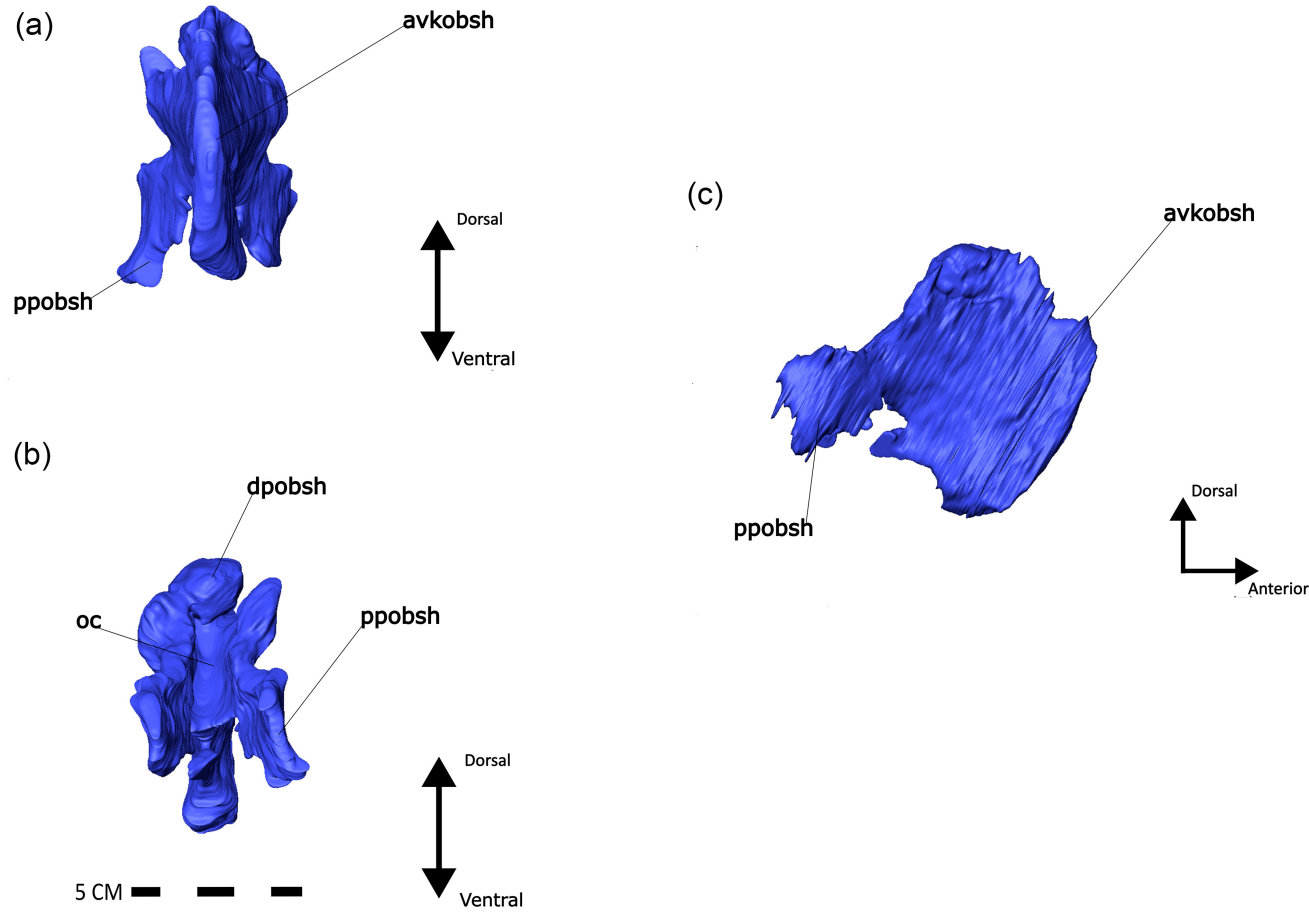
Orbitosphenoid of AM 4950. (a) Anterior view. (b) Posterior view. (c) Right lateral. avkobsh, anteroventral keel of the orbitosphenoid; dpobsh, dorsal process of the orbitosphenoid; oc, olfactory canal; ppobsh, posterior process of the orbitosphenoid. Scale bar equals 5 cm.

The orbitosphenoid is situated between the orbits along the skull's sagittal plane (Figures 1b and 3). It sits mostly free within the cranial cavity. The orbitosphenoid's dorsal process sits within the ventral fossa of the frontal. The anterior face forms an anteroventral keel (Figure 21). The keel is mediolaterally narrow and dorsoventrally tall. The dorsal process of the orbitosphenoid is a rounded knob‐shaped process located dorsally to the olfactory canal. The olfactory canal is located on the posterior side of the orbitosphenoid, between the two posterior processes (Figure 21b). The posterior processes of the orbitosphenoid form lateral extending wings. In lateral view, these wings project in a posteroventral direction.

### 3.29 Dentary

**FIGURE 22:**
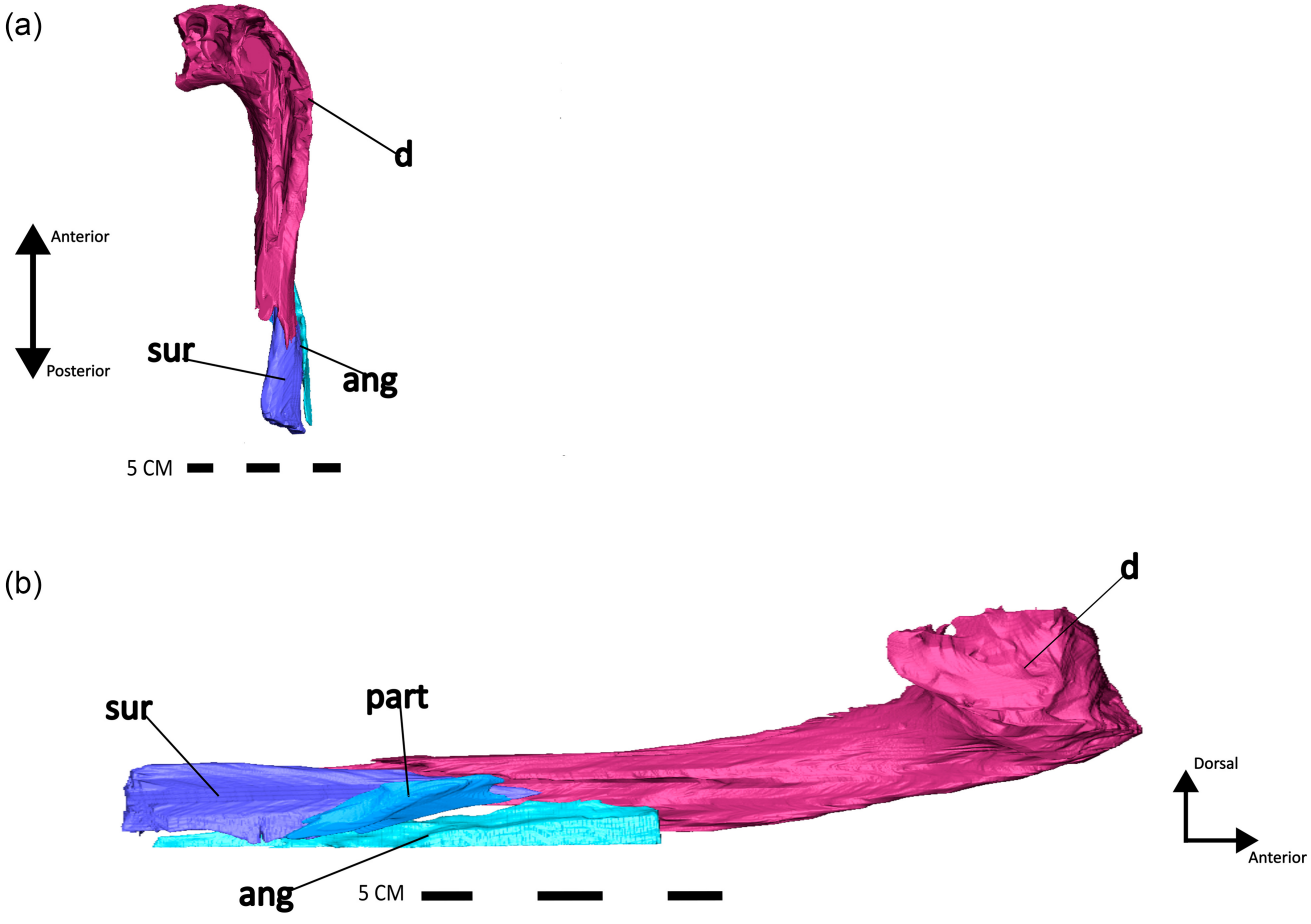
Right mandible of AM 4950. (a) Dorsal view. (b) Medial view. ang, angular; d, dentary; part, prearticular; sur, surangular. Scale bars equal 5 cm.

The dentary is the largest and longest bone in the mandible (Figures 1b and 22). It forms the anterior half of the mandible. In dorsal view, the dentary increases in thickness toward the symphysis (Figure 22a). It sutures the surangular, prearticular, and angular posteromedially. None of the sutures are interdigitated. The posteromedial side overlaps the surangular, prearticular, and angular (Figure 22). The anterior end projects anterodorsally, which gives the mandible a noticeable prognathism. In dorsal view, the dental alveoli form a sulcus (trench) that runs three‐fourths of the length of the dentary. There are at least 14 erupted teeth within the dentary. The posterior surface is irregular. It tapers to a posterior projecting point.

### 3.30 Angular

The angular is thin, elongated, and forms the posteroventral portion of the mandible (Figures 1b and 22). It sutures the dentary anterolaterally, the prearticular dorsomedially, and the surangular posteromedially. None of the sutures are interdigitated. The angular overlaps the prearticular and the surangular. The anterior, dorsal, and posterior edges are irregular. The ventral surface appears flat due to the position of AM 4950 during the CT‐scanning process.

### 3.31 Surangular

The surangular is a thin, elongated structure and forms the posterodorsal portion of the mandible (Figures 1b and 22). It sutures the dentary anterolaterally, the prearticular anteromedially, and the angular ventrolaterally. The surangular is overlapped by the dentary and angular labially and by the prearticular anterolingually. The surangular overlaps the prearticular. The dorsal side is smooth and has a slight medially projecting shelf that roofs part of the adductor fossa. Both the anterior, ventral, and posterior edges are irregular.

### 3.32 Prearticular

The prearticular is a thin and irregularly shaped (Figure 22b). It sutures the dentary and surangular anterolaterally and the angular ventrolaterally. None of the sutures are interdigitated. The prearticular overlaps the dentary, angular, and surangular lingually. The prearticular's anterior, ventral, dorsal, and posterior edges are irregular.

## 4 DENTITION

**FIGURE 23:**
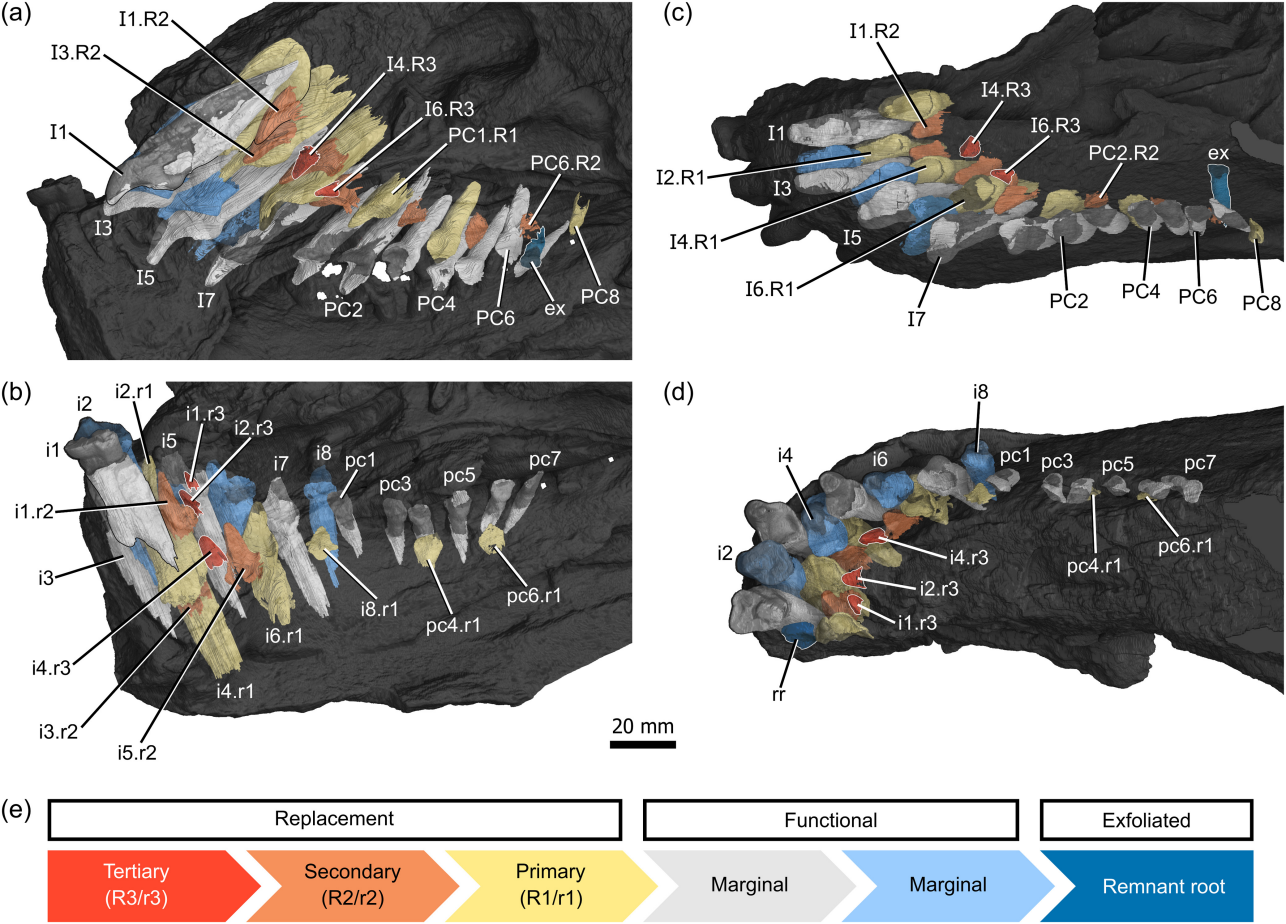
Three‐dimensional renderings of the dentition of a subadult *Moschognathus whaitsi* (AM 4950). Medial views of the right upper (a) and lower (b) dentition. Occlusal views of the right upper (c) and lower (d) dentition. Key to colors used to differentiate between various tooth generations, as well as illustrating the developmental trajectory of the dentition (e). Functional teeth in white and light blue to aid interpretation of alternating replacement pattern in the incisiform teeth. Successive replacement generations (R1–R3) in yellow (primary), orange (secondary), and vermillion (tertiary). Remnant roots and ex situ teeth in blue. ex, ex situ postcanine; I*n*, upper incisiform; i*n*, lower incisiform; PC*n*, upper postcanine; pc*n*, lower postcanine; rr, remnant root. Scale bar equals 20 mm.

**FIGURE 24:**
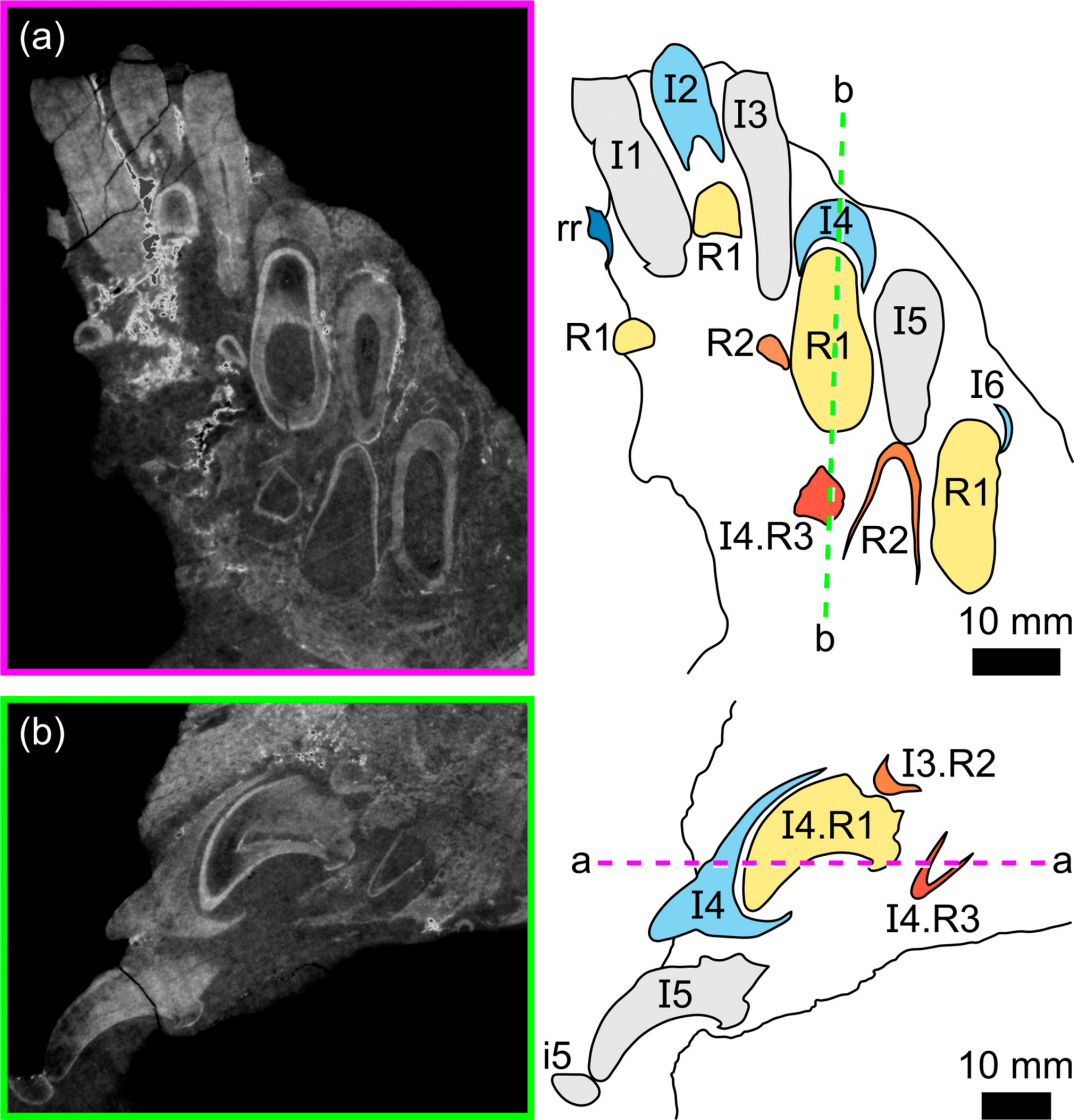
Virtual sections with interpretative drawings through the upper dentition of *Moschognathus whaitsi* (AM 4950). Horizontal (a) and sagittal (b) sections through the fourth upper incisiform (I4). Functional teeth in white and light blue to aid interpretation of alternating replacement pattern. Successive replacement generations (R1–R3) in yellow (primary), orange (secondary), and vermillion (tertiary) to indicate successive generations, and remnant root in blue. I*n*, upper incisiform; i*n*, lower incisiform; rr, remnant root. Scale bars equal 10 mm.

**FIGURE 25:**
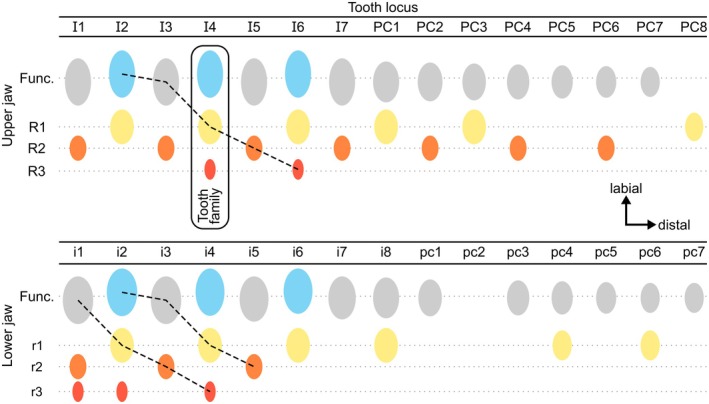
Schematic diagram of the upper and lower dentition of *Moschognathus whaitsi* (AM 4950) explaining the notation used in the text. Func., functional tooth row; I*n*, upper incisiform position; i*n*, lower incisiform position; PC*n*, upper postcanine position; pc*n*, lower postcanine position; R*n*, upper replacement generation; r*n*, lower replacement generation. Three possible examples of *Zahnreihe* are indicated with dashed lines. Note that the indicated upper *Zahnreihe* is the only example to include all five tooth states, whereas the two lower *Zahnreihen* indicated each contain only four tooth states, missing the oldest and youngest tooth, respectively.

Due to the almost homodont nature of the dentition in most tapinocephalid dinocephalians (i.e., lack of an enlarged canine distinguishable from the other dentition of either the maxilla or dentary) (Rubidge, 1991), a modified notation for the dentition is used. To better facilitate comparison in tooth counts between the upper and lower jaws of AM 4950, the teeth are labeled according to crown morphology (Figure 23), rather than the bone bearing them as suggested by Smith and Dodson (2003) for non‐mammalian tetrapods. Due to the association of multiple replacements at each incisiform tooth family, a notation similar to that used by D'Emic et al. (2013) to identify replacements belonging to different generations is adopted (Figures 23, 24, 25).

### 4.1 Upper teeth

Fifteen teeth make up the upper row of functional teeth (Figure 23a, c). Based on crown size and morphology, these can be divided into seven incisiform (I1–I7) and eight postcanine (PC1–PC8) teeth. The five incisiform teeth of the premaxilla have the distinctive “talon and heel” crown morphology associated with tapinocephalid dinocephalians. The eight postcanines (PC1–PC8) are noticeably smaller than the incisiform teeth. The first two teeth of the maxilla (I6–I7) have a rudimentary talon and heel crown morphology similar to the incisiform teeth of the premaxilla, whereas the seven distal teeth (PC1–PC8) have leaf‐shaped crowns (Figure 23a). There is a small diastema between I7 and PC1 (Figure 23c).

The crowns of the upper incisiforms are compressed mesio‐distally, such that the bucco‐lingual length is almost twice that of the mesio‐distal breadth. The crown morphology of these teeth is consistent, but there is a reduction in size toward the distal elements. The first three upper postcanines (PC1–PC3) are larger than the successive teeth (PC4–PC8) and have a different crown morphology. These larger postcanines have a talon and heel, albeit with the angle between the heel and talon being more acute than in the incisiforms. In the four distal postcanines (PC4–PC8) the talon and heel become gradually reduced in size such that they are almost absent in PC6–PC8. As such, the tooth crowns of these distal postcanines appear more spatulate (Figure 23a, b).

Nearly all 14 upper loci with a functional tooth have an associated replacement developing lingually. The exceptions are PC3 (associated replacement seemingly positioned between PC3 and PC4; Figure 23a, b), PC5 (no replacement), and PC7 (no replacement; however, a displaced tooth is present lingual to the functional tooth; Figure 23a, b). Several incisiform teeth have a second replacement positioned lingual to the first. The positioning of these successive replacements, with the talon of the replacement sitting inside a resorption pit in the root of the preceding tooth (Figures 2 and 23c, d) is reminiscent of the complex dental batteries seen in many herbivorous dinosaurs. By contrast, the postcanine dentition has only a single replacement at several loci, with the distal‐most postcanine (PC8) instead having only a developing tooth that was likely still in the process of erupting (Figure 23a, c). Although not a replacement tooth (as there is seemingly no predecessor), PC8 is developmentally similar to those of the primary replacement generation (R1) in that it has a fully formed crown and partially developed root.

The replacement teeth can be subdivided into three generations based on developmental stage and position relative to adjacent teeth. The first replacement generation (R1) has a fully formed crown and partial root. In these teeth, the development of the neck and root ranges from being less than half the length of the talon (e.g., I1.R1 and PC1.R1) to approximately twice the length of the talon (e.g., I2.R1, I4.R1, and I6.R1). The more developed replacement teeth are associated with functional teeth occupying the even‐numbered loci. In all examples, the tip of the talon of the replacement R1 lies within the root of the functional tooth it is to replace. Elements of the second replacement generation (R2) are positioned adjacent to those of R1 (Figures 23 and 24), that is, they occupy odd‐numbered loci. These teeth are less developed than those of the first replacement generation and are mostly represented by fully formed talons. Additionally, some teeth exhibit the partially formed lateral ridges of the developing “heels” (e.g., I5.R2) whereas the less developed teeth have only the beginning of the lingual cingulum (e.g., I3.R2). Unlike the first generation, the crowns of these teeth do not pierce the walls of the functional teeth; however, in some instances, resorption of the roots of the functional teeth is evident (e.g., I3, I5, and I7). The teeth of the third replacement generation (R3) show an even earlier developmental stage than R2, with only the tip of the talon being mineralized. These replacements are positioned in even‐numbered loci, lingual to the elements of the first replacement generation, and do not contact the preceding R1 teeth. Tooth families at even‐numbered loci contain three tooth generations (functional, R1 and R3), whereas odd‐numbered tooth families have only two elements (functional and R2).

### 4.2 Lower teeth

The dentary contains evidence for 15 teeth, which can be separated, based on size and crown morphology, into two groups: eight incisiforms (i1–i8) and seven postcanines (pc1–pc7) (Figure 23b, d). Unlike the upper jaw, there is no diastema between the incisiform and postcanine dentition (Figure 23b, d). There is a gap, large enough to have possibly once held a postcanine tooth, between the first two preserved functional postcanines. This gap does not appear to be an empty alveolus, but it cannot be ruled out that alveolar bone had grown to close the socket, and therefore consider this position to be pc2 (Figure 23b, d). Furthermore, this gap is not identified as a diastema between the incisiform and postcanine tooth groups, as there is a marked difference in size between the teeth interpreted as i8 and pc1 (Figure 23b, d). Finally, the crown morphology of pc1 matches that of other teeth of the postcanine series, supporting the interpretation of pc2 being represented by an empty locus. Although there are six erupted postcanines, our interpretation of the gap as pc2 expands this to seven.

As in the uppers, the row of functional teeth is arranged in a staggered pattern. Replacement teeth are positioned lingual to the functional teeth for all incisiform teeth, whereas only two postcanines (pc4 and pc6) have an associated replacement (Figure 23b, d). As in the upper jaw, the replacement teeth of the dentary can be divided into three generations (r1–r3). Elements of the first replacement generation (r1) occupy positions lingual to the even‐numbered loci, with the second generation in the adjacent odd‐numbered loci. The even numbered incisiform tooth families (i2, i4, i6) comprise of three teeth (functional, r1, r3).

The heels of the lower incisiforms have lateral ridges that flare outwards such that the mesio‐distal breadth (measured at the broadest point) is almost as long as the bucco‐lingual length. The crown morphology of the first seven incisiforms is consistent and shows a gradual reduction in size toward the distal elements, with the distalmost tooth (i8) being considerably smaller than the preceding i7 (Figure 23b, d). Unlike the upper postcanines, all lower postcanines are of a similar size and crown morphology.

The crowns of the replacement lower postcanines (pc4.r1 and pc6.r1) are well‐developed, such that several cuspules are visible along the mesial‐distal margin of the tooth. These cuspules are not visible on the erupted lower postcanines. It is uncertain if the absence of these is due to natural tooth wear while the animal was still alive, or due to postmortem damage either prior to (e.g., weathering) or after fossilization (e.g., damage during excavation or mechanical preparation). Nevertheless, the replacements show that the postcanines are distinguishable from the incisiforms based on both size and crown morphology.

### 4.3 Ex situ teeth

**FIGURE 26:**
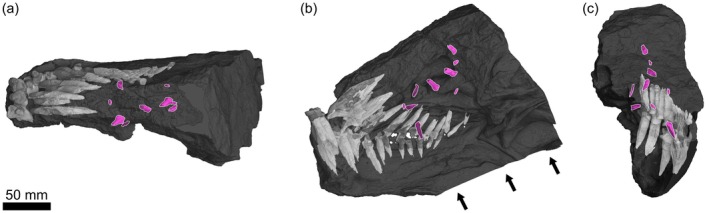
Three‐dimensional rendering of the snout of *Moschognathus whaitsi* (AM 4950) showing positions of the ex situ teeth. Dorsal (a), medial (b), and occipital (c) views. Functional teeth shown in white, ex situ teeth in purple (thin outline). Replacement teeth omitted for better legibility. Arrows indicate missing portion of the mandible due to this region being outside of the beam during scanning. Scale bar equals 50 mm.

In addition to the teeth of the upper and lower jaw preserved in situ (Figure 23), there are several loose teeth preserved in the nasal cavity of AM 4950 (Figure 26). These 10 teeth are of similar crown morphology to the in situ upper and lower postcanines but are of a size more comparable to those of the in situ postcanines of the lower jaw. As there are no empty alveoli present in the right upper, and only a single space in the right lower jaw (pc2; Figure 23b), it is likely that these teeth originated from the postcanine series of the lower left jaw. This interpretation is further supported by the positions of these teeth lingual to the in situ postcanine dentition of the right maxilla (Figure 23a, b).

### 4.4 Tooth replacement rate

**FIGURE 27:**
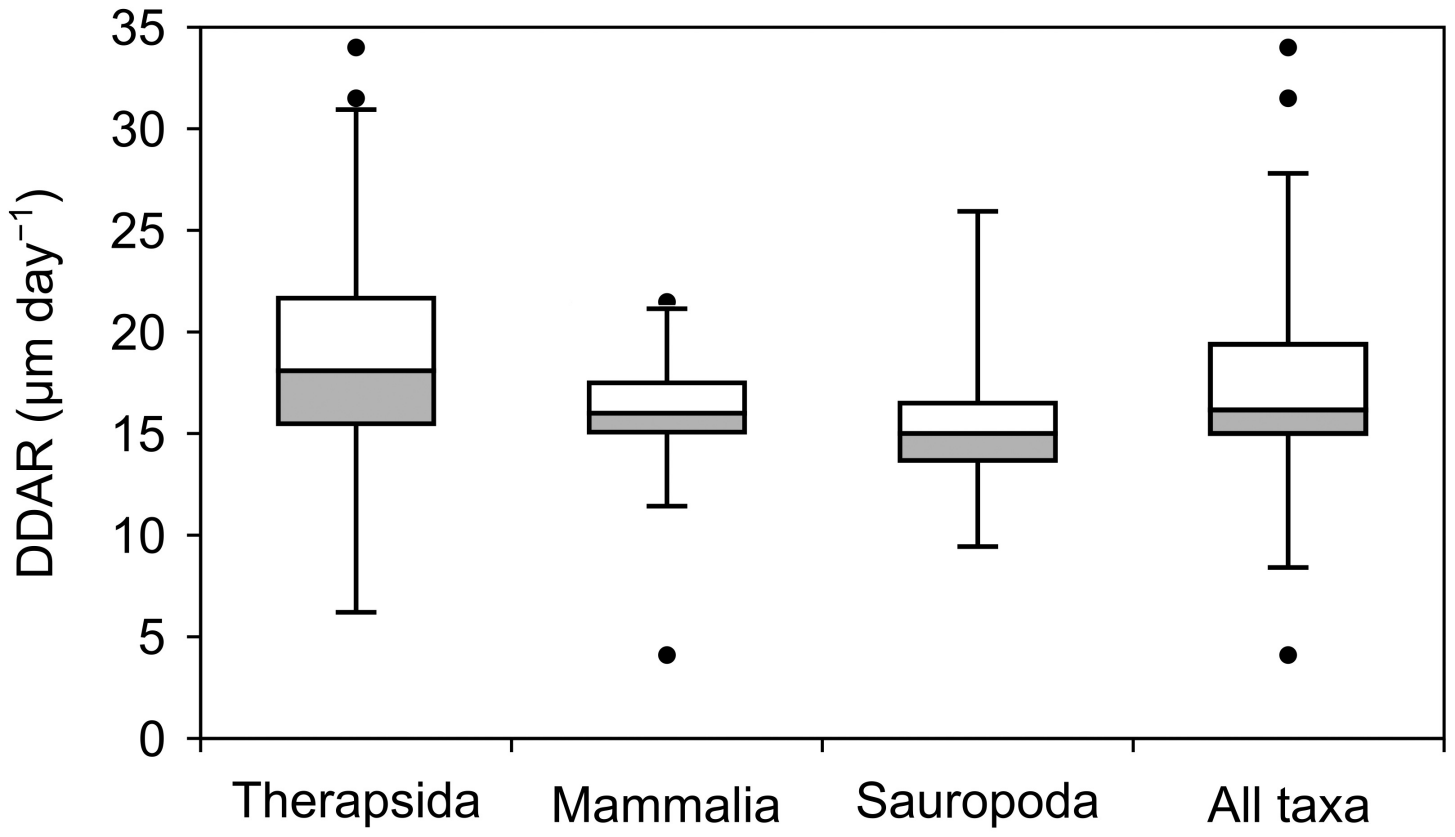
Box plots of widths of incremental growth in the dentine of taxa comparable to *Moschognathus whaitsi* (AM 4950). Error bars indicate the upper and lower limits for outliers (based on 1.5 × interquartile range), with outliers indicated by circles.

Average incremental growth lines were calculated for several taxa, which were separated into three groups: non‐mammalian therapsids, mammalian megaherbivores, and sauropod dinosaurs (Table 2). Using the 1.5 IQR rule (Zar, 2013), possible outliers were identified in non‐mammalian therapsids and mammalian megaherbivores. Within non‐mammalian therapsids, the values for indeterminate tapinocephalid (34 μm) and indeterminate dicynodont (NCSM 21735; 31.5 μm) were identified as outliers greater than the acceptable upper limit (Figure 27). The elevated DDAR of 31.5 μm day^−1^ in the indeterminate dicynodont NCSM 21735 could be due to the measurements having been taken on tusks. It has been demonstrated that, at least in a population of extant African elephants (*Loxodonta africana*), the daily rate of dentine deposition increases with maturity, leading to greater accumulation of mass in the tusks of males in adulthood (Whyte & Hall‐Martin, 2018). However, it must be noted that the converse has been reported in the extinct dwarf elephant *Palaeoloxodon falconeri*, with slower dentine deposition rates reported in mature specimens than in younger specimens (Köhler et al., 2021).

For mammalian megaherbivores, *P. falconeri* (4.1 μm) was identified as falling below the range of accepted values, and *Coryphodon* sp. (21.48 μm) as above the acceptable range (Figure 27). However, it must be noted that when the datapoint for *P. falconeri* is excluded from the sample, the value for *Coryphodon* sp. is not returned as an outlier. By contrast, when *Coryphodon* sp. is removed, the value for *P. falconeri* is still returned as an outlier. For this reason, only the value for *P. falconeri* was considered to represent an outlier. The reduced DDAR in *P. falconeri* is likely due to dwarfism (Köhler et al., 2021). No outliers were identified in the sauropod dinosaur sample. A DDAR was calculated by dividing the difference in dentine thickness between successive generations of the same tooth family (Table 3) by the average dentine incremental line thickness (Table 2). Two values are presented for non‐mammalian therapsids and mammalian herbivore samples; the first encompassing the complete sample and a second with outliers removed.

With outliers removed, the average dentine incremental line thickness for the three groups was: non‐mammalian therapsids, 16.93 μm; mammalian megaherbivores, 16.65 μm; and sauropod dinosaurs, 15.18 μm. These values closely matched the average daily incremental growth in dentine of ~16 μm in vertebrates identified by Schour and Hoffman (1939). Despite the incremental line distance of 34 μm given by Whitney and Sidor (2019) representing an outlier when compared to the rest of our sample (Table 1), this is currently the only dentine thickness for a tapinocephalid dinocephalian reported in the literature. Therefore, in addition to using the average DDARs with outliers removed (see Table 2) to calculate a range of replacement rates for the dentition of AM 4950, a range of replacement rates using a DDAR of 34 μm day^−1^ was also calculated for comparison (Table 4).

The difference in dentine thickness between the functional and primary replacement was consistently thinner than the difference in thickness between the functional and secondary replacement (Table 4). In the incisiforms, there is a greater average difference in dentine thickness between the functional and secondary replacement of odd‐numbered loci than the average difference observed between the functional and primary replacement in even‐numbered loci. No such trend is evident in the postcanines, as all replacements were considered to belong to the primary replacement generation. This apparent thinning of the dentine between adjacent functional teeth may be due to resorption of the pulp cavity caused by the development of the replacing tooth. As the dentition of the primary replacement generation is more developed than that of the secondary generation (Figure 23), it is likely that the functional teeth with associated primary replacements have undergone more resorption, resulting in a thinner dentine layer.

Although several tooth replacement rates were calculated for AM 4950 based on five hypothetical DDARs (Table 4), for brevity only the range of estimated tooth replacement rates calculated using the highest and lowest average DDAR, and the indeterminate tapinocephalid of Whitney and Sidor (2019) will be described in detail. Interestingly, for all three tooth groups (representative tooth, average incisiform, and average postcanine) the range of replacement rates calculated for the lower dentition was below the ranges for the upper dentition.

The lowest DDAR in our sample (sauropod dinosaurs, 15.18 μm day^−1^) returned a tooth replacement rate ranging from 38 to 256 days (median of 131 days), whereas the highest DDAR (non‐mammalian therapsids, 16.93 μm day^−1^) gave a range of 34 to 229 days, with a median of 118 days. Being more than double the average value for non‐mammalian therapsids, the DDAR of 34 μm day^−1^ for an indeterminate tapinocephalid returned a replacement rate of 17–114 days, or approximately half that calculated using the DDAR of non‐mammalian therapsids and sauropod dinosaurs, with a median of 59 days. It must be noted that both the taxonomic identification and ontogenetic age of the indeterminate tapinocephalids studied by Whitney and Sidor (2019) are uncertain, such that the average DDAR, in isolation, may not be accurate with regard to estimating the tooth replacement in AM 4950. Although Whitney and Sidor (2019) measured a range of dentine thicknesses in indeterminate tapinocephalids, they did not report how many incremental lines were counted in each tooth. The median of all calculated values gives an estimated replacement rate of 108 days.

## 5 DISCUSSION

### 5.1 Potential adaptations to headbutting

Headbutting has been seen as a parsimonious explanation for the high degree of pachyostosis found in tapinocephalids (Barghusen, 1975; Benoit, Manger, et al., 2017; Geist, 1972). Brink (1958) was the first to propose headbutting in a tapinocephalid dinocephalian. This idea was later expanded to include intraspecific competition (Barghusen, 1975; Geist, 1972). It was later hypothesized that Tapinocephalia primarily used headbutting as a form of intraspecific competition, whereas Anteosauria primarily used their teeth (Benoit et al., 2026; Benoit, Kruger, et al., 2021; Benoit, Manger, et al., 2017). The evidence for direct headbutting in tapinocephalians like *Moschops, Criocephalosaurus*, and *Moschognathus* is more plausible due to the high potential contact surface around the frontal–parietal region (Barghusen, 1975; Kemp, 2005). The ventrally tilted skull, the system of large postorbital bars and post‐temporal arches supporting the dorsal head shield, extensive pachyostosis to protect the central nervous system, and re‐orientation of the braincase to transfer the energy of impact to the neck are all characteristics potentially consistent with headbutting (Barghusen, 1975; Benoit, Manger, et al., 2017).

Observations of AM 4950 that may serve as adaptations to headbutting include a vertically thick frontal and parietal, a medially thick postfrontal, an enlarged prefrontal, a laterally thick postorbital, a compact pterygoid, a robust orbitosphenoid, and the presence of a paroccipital, which is the fusion of the prootic, opisthotic, and exoccipital. The sutures within the paroccipital are indistinguishable, especially when compared to other sutures in the skull. It is, therefore, proposed that the bones forming the paroccipital are some of the first to completely fuse in ontogeny. Asynchronous fusions of cranial bones have been documented in mammals, with a correlation between encephalisation quotients and the ossification of endocranial bones (Koyabu et al., 2014). It was found that mammals with a higher encephalisation quotient have a more rapid ossification of the supraoccipital (Koyabu et al., 2014). While Benoit, Manger, et al. (2017) analysis of AM 4950's endocasts shows that it had a surprisingly high encephalisation quotient for a Permian therapsid, it would be an exaggeration to infer that this slight excess of encephalisation would justify the extreme pachyostosis observed in dinocephalians. More work is needed before any homologies between mammalian and non‐mammalian therapsid cranial ossification can be considered.

It is worth discussing the scarcity of interdigitated sutures in *Moschognathus*. If placed on the suture sinuosity index (White et al., 2020), AM 4950 sutures would rank near one (the lowest). Interdigitated sutures are believed to correlate with a higher tolerance to compressive forces (Jaslow, 1989; Kammerer, 2021). Jaslow (1989) demonstrated that male wild sheep (*Ovis orientalis*) have a higher cranial suture complexity than females. Male wild sheep practice headbutting as a form of interspecific competition (Jaslow, 1989). However, in the absence of interdigitated sutures, an expansion of surface area has been shown to work equally well (Dudgeon & Evans, 2023). The large surface area of the frontal‐parietal shield in *Moschognathus* fits this second adaptation. The scarcity of interdigitated sutures is also seen in other tapinocephalid skulls such as *Tapinocaninus*, *Moschops*, *Avenantia*, and *Riebeeckosaurus* (Güven et al., 2013; Neumann, 2020; Rubidge, 1991). As such, it can be hypothesized that the large skull of tapinocephalids increased surface area for fights and fusion of sutures, hence compensating for the absence of suture complexity. Despite AM 4950's pachyostosis, the sutures between the bones had not yet completely fused (suggesting a subadult state), making the margins the most vulnerable areas of the skull.

### 5.2 Frontal bone abscess

Paleopathologies are important when trying to assess the behavior of extinct organisms, such as bone lesions in gorgonopsians, an embedded tooth in a gorgonopsian snout, and a dicynodont cranial cyst (Benoit, Browning, & Norton, 2021; Kato et al., 2020; Vega & Maisch, 2014). The abscess of specimen AM 4950 was described in Benoit et al. (2026). The high bone density around the margins of the abscess provides direct evidence for healing and so excludes post‐mortem agents. The location of the abscess within the tripartite suture (between the frontal, prefrontal, and postfrontal) may have slowed down the healing process (Figure 10). The abscess's internal location excludes any external causes, such as a bite from a predator. Finally, the presence of drainage canals along the margins of the abscess hints toward an infection (Figure 10b). The direct cause for this injury is hypothesized to be either from single or multiple frontal bone impacts; the abscess's location beneath the most likely impact area helps support this hypothesis.

Among modern genera, adult male Bighorn Sheep (*Ovis canadensis*) have an average skull length of 30 cm (the same as AM 4950) and can generate an impact force of around 3400 N (Kitchener, 1988; Rutter et al., 1972). For reference, it takes around 1600 N to break a human femur (Cummins et al., 2011). Despite the adaptations for and documentation of headbutting in modern genera, cranial injuries are still common (Ackermans et al., 2021, 2022). Injury severity variation is dependent on both age and sexual dimorphism. For example, the male muskoxen (*Ovibos moschatus*) skull is 300% larger than the females (Ackermans et al., 2021). However, Ackermans et al. (2022) findings show that despite the reinforcements in male muskoxen skulls, brain trauma is still common.

Although the mechanics behind headbutting in bovids and *Moschognathus* may differ, these previously stated measurements provide a baseline estimate of the impact force *Moschognathus* may have been capable of producing, which may support the hypothesis that headbutting was the likely cause of this pathology. Hydraulic pressure experiments will be necessary to estimate how much force would have been required to create such a wound.

**FIGURE 28:**
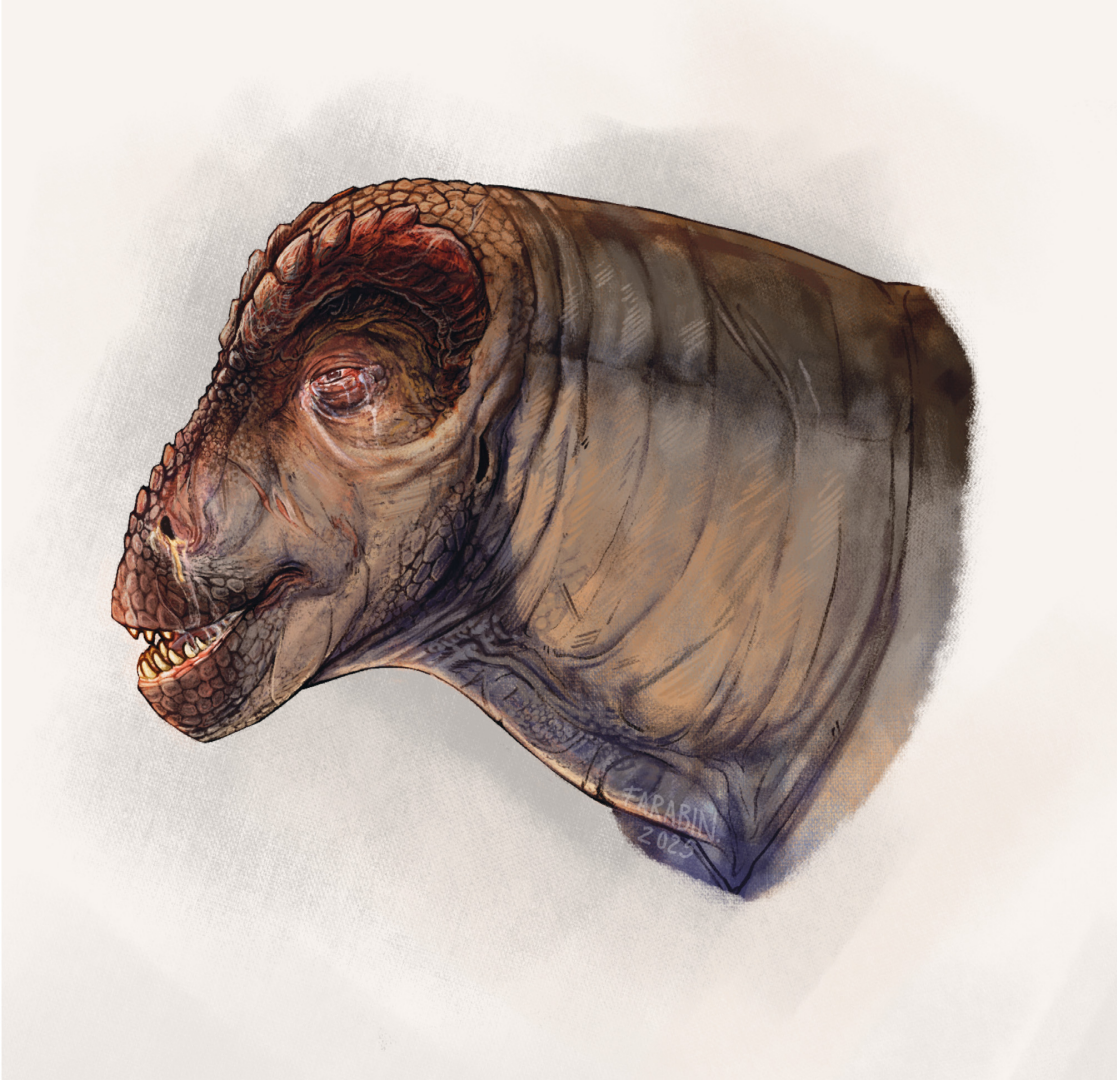
Life reconstruction of juvenile *Moschognathus whaitsi* (AM 4950) in lateral view. Note the fluid running from the eyes and nostrils. Artist: Morgan Farabin.

Whether or not this injury sustained by AM 4950 was enough to contribute to its death is difficult to estimate. The margins of healing around the abscess indicate that the animal lived long enough for some degree of healing to occur (Figure 10). However, the abscess opens behind the right orbit and sinus cavity, which may have led to the spread of infection (Figure 28). If an infection had spread into the sinus cavity and the right orbit, it may have impacted the animal's lifespan. The location of the abscess does not suggest any direct injury to the brain itself. However, if the infection did manage to spread to the brain, death would have been highly probable. While there are many documented differences in the extant mammalian and reptilian immune systems (Zimmerman, 2020), it is unknown whether these differences extend to the broader synapsid and sauropsid clades. It is, therefore, difficult to determine whether the injury sustained by AM 4950 was fatal.

### 5.3 Cranial and dental adaptations for herbivory

The peculiar “talon‐and‐heel” crown morphology typical of tapinocephalid dinocephalians has long been regarded as an adaptation toward herbivory. The talons form a comb‐like row in the front of the mouth, comparable to the dentition of various sauropod dinosaurs, particularly the narrow‐crowned and peg‐like teeth of diplodocoids and titanosaurs (Button et al., 2017; Salgado & Calvo, 1997; Young et al., 2012). However, unlike narrow‐crowned sauropods where the dentition is concentrated in the anterior of the rostrum (Christiansen, 2000; D'Emic et al., 2013; Young et al., 2012), the tooth row of *Moschognathus* extends distally along most of the jaw (Figure 1a). In this regard, the dentition of AM 4950 more closely resembles that of broad‐crowned sauropods (e.g., *Camarasaurus* and *Brachiosaurus*) (Button et al., 2016; Christiansen, 2000; D'Emic et al., 2013), which tend to have a more robust skull, a shorter snout, and a longer tooth row than narrow‐crowned sauropods (Button et al., 2017; Christiansen, 2000; D'Emic et al., 2013). In lateral view (Figure 1), the skull proportions of *Moschognathus* more closely resemble that of the narrow‐crowned *Diplodocus*, whereas the tooth row extending along the jaws is closer to the condition of the broad‐crowned *Brachiosaurus*. Thus, *Moschognathus* has a mosaic of features shared between both narrow‐ and broad‐crowned sauropod dinosaurs.

Pachyostosis of skull roof in tapinocephalid dinocephalians is often regarded as an adaptation toward headbutting behavior (e.g., Barghusen, 1975; Benoit, Manger, et al., 2017; Brink, 1958); the increased surface area surrounding the temporal fenestrae may also have facilitated an increased area for attachment of masticatory muscles (Kemp, 1982). A similar reduction in size of the supratemporal fenestrae in some sauropod dinosaurs has been attributed to the rearrangement of the jaw musculature (Button et al., 2017; Sereno et al., 2007).

The specialized teeth of sauropod dinosaurs have long been hypothesized to have been used to strip foliage from branches in a rake‐like fashion, using either the upper or lower teeth independently, or using them together (Barrett & Upchurch, 1994; Young et al., 2012). Christiansen (2000) noted that, at least for *Diplodocus*, the slender teeth may have been unsuitable for the stresses associated with this type of feeding, suggesting that more anteriorly protruding dentition would be better suited for such a feeding mechanism. Analysis of microwear features on the dentition of *Diplodocus* (Whitlock, 2011) revealed the presence of pits, rather than striations, which are more commonly associated with branch‐stripping behaviors (e.g., Ryan, 1981). Based on these results, Whitlock (2011) argued that *Diplodocus* was likely a low‐level browser. Nonetheless, biomechanical modeling of the skull of *Diplodocus* has demonstrated that branch‐stripping was a feasible feeding mechanism (Young et al., 2012), even if this was not the primary feeding behavior.

Unlike the dentition of sauropod dinosaurs, the anterior dentition of tapinocephalid dinocephalians features a prominent “heel” or lingual cingulum (Figure 2). The incisiforms of AM 4950, especially the lowers, bear prominent lateral ridges that flank the heel, forming a lingual depression (Figure 23c, d). It is thought that these ridges formed a cutting edge. The talons of the upper and lower teeth are often interpreted to have intermeshed during jaw adduction, such that the heels/cingula of opposing teeth in the upper and lower jaw came into contact to form a crushing/shearing surface (Boonstra, 1962; Whitney & Sidor, 2019). This intermeshing of the dentition was thought to be a distinguishing character uniting all Dinocephalia (Boonstra, 1962); however, intermeshing of the incisors has also been described in Biarmosuchia (Rubidge et al., 2006; Sidor & Welman, 2003). In AM 4650, the lower jaw protrudes rostrally, such that the lower incisiforms no longer occlude with the uppers (Figure 1a). Similarly, it seems that the lower postcanines would have passed lingual to the uppers during occlusion. This is similar to the condition described by Boonstra (1962) for titanosuchid dinocephalians, and Rubidge (1991) for the basal‐most tapinocephalid *Tapinocaninus pamelae*. The procumbent orientation of the incisiform teeth in AM 4950 (Figure 23) may have facilitated a comb/rake‐like feeding mechanism. However, rather than the lateral ridges forming a precise cutting surface against opposing teeth, they may have been used to shear plant material in a rake‐like manner.

Use of the dentition in this manner often leaves distinct wear marks on the tooth crowns (Fiorillo, 1998; Whitlock, 2011). The presence of microwear on the functional dentition of AM 4950 cannot be determined as (a) the occlusal surfaces are mostly obscured due to the lower jaw being tightly occluded (Figure 1); (b) dental microwear features in herbivorous dinosaurs typically have a width between ~2 and 27 μm (Mallon & Anderson, 2014; Whitlock, 2011), therefore any microwear features potentially preserved on the dentition of AM 4950 would likely be considerably smaller than the 91 μm voxel size of the SRXCT data. As such, there is no visible difference between the tooth crown surfaces of the erupted functional tooth and the associated replacement (Figure 23). Although the width of microwear features has been considered to sometimes be uninformative by several researchers (Goswami et al., 2005; Teaford & Runestad, 1992), if present, being able to quantify the orientation and frequency of any microwear features would possibly provide further insight into the diet of *Moschognathus*.

Whitney and Sidor (2019, figure 2c–e) produced a model of the tooth‐on‐tooth occlusion, depicting intermeshing of the talons and a precise occlusion between the lateral ridges of the upper and lower teeth. This representation has several problems. Whitney and Sidor's (2019) model relies on cloning the model of a single tooth several times and does not take into account the variation in crown size and shape differences between the upper and lower teeth evident in the nearly complete dental series preserved in AM 4950 (Figure 23). Similarly, their model assumes that the teeth were aligned along the margin of the premaxilla and dentary in a uniform manner, allowing for precise occlusion. The dentition of AM 4950 instead shows a distinct alternation in alignment, likely due to the functional row being composed of two (or more) tooth generations, with odd‐numbered upper and lower incisiforms protruding slightly labial compared to the adjacent even‐numbered teeth (Figure 23c, d). However, we are uncertain whether the staggered appearance of the dentition has been accentuated due to the partial exfoliation of the teeth during preservation. The teeth of dinocephalians are typically attached by a soft tissue gomphosis, rather than a bony fusion to the jaw (ankylosis) as seen in several other groups of therapsid (LeBlanc et al., 2018). This gomphosis was likely an adaptation to facilitate a slight movement of the dentition to better maintain a precise occlusion between the upper and lower tooth crowns during jaw adduction. Finally, the reconstructed teeth in Whitney and Sidor's (2019) model (particularly the uppers) are not as procumbent as those observed in AM 4950 (Figures 1a, 23, and 26).

Examination of the alignment of the positions of the marginal tooth crown in AM 4950 could demonstrate that the feeding mechanics of tapinocephalid dinocephalians were not reliant on a precise occlusion between opposing heels, but rather used the talons of the upper and lower teeth together to strip leaves and shoots from plants, similar to that proposed in sauropod dinosaurs (Barrett & Upchurch, 1994).

Boonstra (1953, p. 21) posed the question “is the heterodont Titanosuchian dentition to be morphologically derived from the homodont dentition of the Tapinocephalians?” Our current understanding of dinocephalian taxonomy instead suggests that the homodont dentition of tapinocephalids were derived from the heterodont dentition of titanosuchid dinocephalians.

The extension of the incisiform teeth onto the maxilla is likely indicative that, rather than the caniniform being lost (as in modern rodents and bovids), the tooth has been reduced in size and taken on a crown morphology comparable to the mesial incisiform. Evidence for this comes in the form of *Tapinocaninus*, which has a reduced maxillary canine (Rubidge, 1991), and has dentition representing a transitional form between basal non‐tapinocephalid dinocephalians and more derived tapinocephalids, including *Moschognathus*.

### 5.4 Tooth replacement

Gregory (1926) suggested that tapinocephalid dinocephalians may have been diphyodont. Ironically, Gregory (1926, plate XX) used the holotype of *Moschognathus* (AMNH FARB 5602) to illustrate his interpretation, whereas, based on our results, we can conclusively demonstrate that *Moschognathus* (AM 4950) was polyphyodont, with multiple replacements present in a single tooth family at several loci in both the upper and lower jaw (Figures 23, 24, 25).

Boonstra (1952) referred to the multiple generations of teeth evident in *Agnosaurus pienaari* (SAM‐PK‐11832) as “milk teeth.” Multiple replacement generations have also been described in the titanosuchid dinocephalian *Jonkeria* (Boonstra, 1962). Boonstra (1969) had previously noted the asymmetry in tooth replacement in dinocephalians. Successive replacement teeth seemingly form rows of *Zahnreihen* (Figure 25), the model of replacement favored by Edmund (1960, 1962); however, see also Osborn (1972) and LeBlanc and Reisz (2015) for comments on how this model may be unsuitable for describing the eruption sequence of replacement teeth in fossil taxa.

The staggered arrangement of the incisiform teeth, when viewed in occlusal view, suggests an alternating pattern of replacement. In contrast to the incisiform dentition, the postcanines show less replacement activity. Not all postcanine loci have an associated replacement, and none have more than one developing replacement (Figures 23 and 25). The staggered arrangement of the functional upper teeth, coupled with the alternating presence of replacement teeth, suggests that at any given time, the row of functional upper teeth comprised at least two generations of differently aged elements.

The presence of multiple replacements associated with the incisiform dentition of *Moschognathus* is reminiscent of the dental batteries in sauropod dinosaurs, albeit millions of years before this had evolved in dinosaurs, and was likely a further adaptation toward a herbivorous lifestyle. The positioning of a dental battery in the rostral/incisiform region of the mouth is also present in sauropod dinosaurs and is taken to the extreme in the rebbachisaurid *Nigersaurus taqueti*. A dental replacement rate of ~30 days was estimated for *Nigersaurus* (Sereno et al., 2007); however, a more recent study produced a much higher estimate of only 14 days (D'Emic et al., 2013). This latter study included several sauropod taxa, resulting in a range of estimated replacement rates spanning from ~14 to 98 days, with *Nigersaurus* (narrow‐crowned) and *Mamenchisaurus* (broad‐crowned) representing the two extremes.

Whitney and Sidor (2019) reported a distance between incremental lines of dentine deposition similar to that of modern mammals. Due to limited sample size, Whitney and Sidor (2019) were unable to provide an estimate of replacement rate using the methods established by Erickson (1996a, 1996b). Higher resolution scans that allowed for the visualization, measurement, and counting of daily incremental lines of dentine in teeth belonging to the same tooth family would be needed to calculate an accurate tooth replacement rate for AM 4950.

Using the data available to them, Whitney and Sidor (2019) tabulated the “proportion of tooth positions in replacement” for several synapsid taxa and used this metric to estimate dental replacement rates. They identified that tapinocephalid dinocephalians had a higher‐than‐average proportion of tooth loci with active replacement when compared to other therapsid taxa. However, a higher proportion of teeth undergoing replacement does not necessarily indicate a higher *rate* of replacement, but rather a higher *degree* of replacement. That is to say, an individual with 80% of the functional teeth having an associated replacement developing will replace a minimum of 80% of its teeth, whereas an individual with only 20% of the functional teeth having replacements could, hypothetically, still replace 20%–100% of its dentition, depending on where the individual is in their ontogenetic trajectory, as well as environmental influence, would impact this percentage. For example, an individual with a lower proportion of tooth loci undergoing replacement may be due to individual either being at an earlier (e.g., only 20% of the teeth show replacement activity as the remaining replacements have yet to develop) or later ontogenetic stage (e.g., only 20% show replacement activity, as other teeth have already undergone replacement). This latter explanation is likely to be more quantifiable in taxa that exhibit a finite number of replacement generations. Similarly, the number of days it takes for a tooth to develop is not necessarily indicative of how long the tooth will occupy the functional position before being shed.

However, using the proportion of actively replacing loci as a proxy for replacement rate is potentially an inappropriate method. For instance, take examples of human infant skulls that have been prepared to show the unerupted deciduous dentition (e.g., Dean & Lucas, 2009). These often show replacement at every functional locus, as well as several additional unerupted teeth that have no functional predecessor (i.e., the molars) per side; if percentages were used, these individuals would score over 100%. This is not to say that the rate of replacement was extremely rapid, as it takes several years for a child to fully replace the deciduous dentition, normally between the ages of ~5 to 13 years. This is followed by a period during which the molars erupt, and in some cases, the third molars (or wisdom teeth) do not erupt. The increased degree of replacement reported in tapinocephalid dinocephalians may be due to an adaptation to continuously replace their dentition throughout ontogeny, that is, they were polyphyodont. Conversely, there is a growing body of work suggesting that theriodont therapsids adapted to reduce the number of tooth replacement generations to a finite number (i.e., were oligophyodont), culminating in true mammalian diphyodonty in the Jurassic (Benoit et al., 2022; Mao et al., 2019; Norton et al., 2020, 2021, 2023).

Studies of tooth replacement in therapsids with distinctive caniniforms have found that these forms have rather conservative replacement patterns in the anterior dentition (incisors + canines), whereas replacement patterns of postcanines show more variation (Abdala et al., 2013; Norton et al., 2020, 2021). The anterior dentition undergoes alternating replacement (plesiomorphic condition) and the number of teeth in the series remains constant through ontogeny (Güven et al., 2013; Norton et al., 2020, 2021). In contrast, the number of teeth in the postcanine series is variable through ontogeny and migrates distally, with the pattern of replacement becoming sequential (Abdala et al., 2013; Brink, 1977; Fourie, 1963; Hopson, 1971; Martinelli & Bonaparte, 2011; Norton et al., 2021). This switch is likely linked to each postcanine locus having a finite number of replacement teeth. Interestingly, despite lacking a clear caniniform tooth (Figure 23), the dentition of AM 4950 can be divided into two groups based on crown morphology. Similarly, when looking at the replacement of these two groups, the anterior “incisiforms” show an increased number of replacement elements (20 replacements associated with 15 loci), whereas the “postcanine” dentition has fewer replacements (8 replacements between 14 loci). There is a marked reduction in the number of replacements associated with the postcanines. This can be quantified by calculating the ratio of functional loci to replacements (primary and secondary generations), with ~93% of the incisiforms undergoing replacement (only i7 lacks an associated replacement), although this figure surpasses >133% if the tertiary generations are also included. By contrast, only ~53% of the postcanine loci were undergoing active replacement, with the uppers showing a higher percentage of replacement (>70%) than the lowers (~29%) (Figures 23 and 25).

A clear alternating pattern is evident in both the upper and lower incisiforms, with the even‐numbered loci having three generations (functional, R1, R3) per tooth family, and the odd‐numbered loci having two generations (functional and R2). This is the same alternating pattern described in more derived theriodont therapsids (Abdala et al., 2013; Kermack, 1956; Norton et al., 2020, 2021). The most notable difference is the increased number of replacements associated with each tooth family.

Taking all of these points into consideration and using the methodology proposed by Erickson (1996b) and a series of measurements of the daily dentine growth increments for extinct and extant taxa taken from the literature (Table 1), it was possible to calculate a range of tooth replacement rates for AM 4950 (Table 4). These replacement rates varied from 17 to 256 days, dependent on which DDAR was used (Table 2), and whether the teeth belonged to the upper versus lower jaw, or incisiform versus postcanine series (Table 4). The seemingly higher replacement rate of the postcanines (17–108 days) compared to the incisiforms (51–256 days) is perhaps a result of there being fewer replacement postcanines (Figure 23, Table 3). The median replacement rate in AM 4950 is 108 days. This is slower than the tooth replacement rate reported by Erickson (1996a) in herbivorous dinosaurs (46–83 days), but is faster than rates measured in carnivorous dinosaurs (290–777 days) from the same study. D'Emic et al. (2013, 2024) expanded the taxon sampling of sauropod dinosaurs and reported a much wider range of tooth replacement rates of 14–98 days. As such, the slower replacement rate of 108 days in *Moschognathus* is more similar to the replacement rate of sauropods with broad‐crowned dentition (e.g., *Giraffatitan*, 92 days; and *Mamenchisaurus*, 98 days) as opposed to the faster replacement rate seen in sauropods with narrow tooth crowns (e.g., *Diplodocus*, 35 days) (D'Emic et al., 2013, 2024).

The combination of a dentition with crushing surfaces concentrated at the front of the mouth, an increased number of replacement teeth, a relatively rapid rate of tooth replacement, and a large body size all point to the tapinocephalids having adapted to a herbivorous diet, likely fulfilling an ecological niche like that of modern mammalian megafauna.

## 6 CONCLUSION

The first 3D analysis of the entire skull of a dinocephalian illustrates the morphology of internal cranial structures and the tooth replacement system within this clade. This results in a better understanding of the anatomy of AM 4950, which has yielded the discovery of some of the first internal pathologies for therapsids and will form the basis of new descriptive works. The analysis of tapinocephalian teeth also illustrates the diversity of therapsid dental morphology and replacement, as well as demonstrating some level of homoplasy between tapinocephalians and sauropod dinosaur dental replacement. Being one of the first complete 3D analyses of a dinocephalian means that interclade comparison is difficult. But with the advancements in synchrotron technology and the acquisition of more specimens, it may be possible to produce a repository of dinocephalian skulls that may be used for both a more holistic descriptive and phylogenetic analysis.

## AUTHOR CONTRIBUTIONS

**Tristen Lafferty:** Writing – original draft; investigation; writing – review and editing; methodology; conceptualization; visualization. **Luke A. Norton:** Investigation; writing – original draft; writing – review and editing; methodology; conceptualization; supervision; resources; visualization. **Aliénor Duhamel:** Writing – review and editing; resources; supervision; validation. **Julien Benoit:** Project administration; supervision; resources; writing – review and editing; conceptualization; validation.

## FUNDING INFORMATION

This research is financially supported by the National Research Foundation through an International Science and Technology Collaboration travel grant (UID 68707), the African Origins Platform (AOP210218587003; AOP240418214774), and the University of the Witwatersrand.

## References

[ar70038-bib-0001] Abdala, F. , Jasinoski, S. C. , & Fernandez, V. (2013). Ontogeny of the early Triassic cynodont *Thrinaxodon liorhinus* (Therapsida): Dental morphology and replacement. Journal of Vertebrate Paleontology, 33, 1408–1431. 10.1080/02724634.2013.775140

[ar70038-bib-0002] Ackermans, N. , Hof, P. , & Reidenberg, J. (2021). Does headbutting cause traumatic brain injury? The case of combative bovids. The FASEB Journal, 35(S1). 10.1096/fasebj.2021.35.S1.03638

[ar70038-bib-0003] Ackermans, N. L. , Varghese, M. , Williams, T. M. , Grimaldi, N. , Selmanovic, E. , Alipour, A. , Balchandani, P. , Reidenberg, J. S. , & Hof, P. R. (2022). Evidence of traumatic brain injury in headbutting bovids. Acta Neuropathologica, 144, 5–26. 10.1007/s00401-022-02427-2 35579705 PMC9217783

[ar70038-bib-0004] Barghusen, H. R. (1975). A review of fighting adaptations in dinocephalians (Reptilia, Therapsida). Paleobiology, 1, 295–311. 10.1017/S0094837300002542

[ar70038-bib-0005] Barrett, P. M. , & Upchurch, P. (1994). Feeding mechanisms of *Diplodocus* . Gaia, 10, 195–203.

[ar70038-bib-0006] Benoit, J. , Araujo, R. , Lund, E. S. , Bolton, A. , Lafferty, T. , Macungo, Z. , et al. (2026). Early synapsids neurosensory diversity revealed by CT and synchrotron scanning. The Anatomical Record, 309, 912–929. 10.1002/ar.25445 PMC12961373 38600433

[ar70038-bib-0007] Benoit, J. , Browning, C. , & Norton, L. A. (2021). The first healed bite mark and embedded tooth in the snout of a middle Permian gorgonopsian (Synapsida: Therapsida). Frontiers in Ecology and Evolution, 9, 699298. 10.3389/fevo.2021.699298

[ar70038-bib-0008] Benoit, J. , Fernandez, V. , Manger, P. R. , & Rubidge, B. S. (2017). Endocranial casts of pre‐mammalian therapsids reveal an unexpected neurological diversity at the deep evolutionary root of mammals. Brain, Behavior and Evolution, 90, 311–333. 10.1159/000481525 29130981

[ar70038-bib-0009] Benoit, J. , Kruger, A. , Jirah, S. , Fernandez, V. , & Rubidge, B. S. (2021). Palaeoneurology and palaeobiology of the dinocephalian therapsid *Anteosaurus magnificus* . Acta Palaeontologica Polonica, 66, 29–39. 10.4202/app.00800.2020

[ar70038-bib-0010] Benoit, J. , Manger, P. R. , Norton, L. , Fernandez, V. , & Rubidge, B. S. (2017). Synchrotron scanning reveals the palaeoneurology of the head‐butting *Moschops capensis* (Therapsida, Dinocephalia). PeerJ, 5, e3496. 10.7717/peerj.3496 28828230 PMC5554600

[ar70038-bib-0011] Benoit, J. , & Midzuk, A. J. (2024). Estimating the endocranial volume and body mass of *Anteosaurus*, *Jonkeria*, and *Moschops* (Dinocephalia, Therapsida) using 3D sculpting. Palaeontologia Electronica, 27, a39. 10.26879/1377

[ar70038-bib-0012] Benoit, J. , Nxumalo, M. , Norton, L. A. , Fernandez, V. , Gaetano, L. C. , Rubidge, B. , & Abdala, F. (2022). Synchrotron scanning sheds new light on *Lumkuia fuzzi* (Therapsida, Cynodontia) from the Middle Triassic of South Africa and its phylogenetic placement. Journal of African Earth Sciences, 196, 104689. 10.1016/j.jafrearsci.2022.104689

[ar70038-bib-0013] Boonstra, L. D. (1952). *Agnosaurus* gen. nov.; ‘n Nuwe geslag van die deinocephaliërs. Tydskrif Vir Wetenskap en Kuns, 12, 242–245.

[ar70038-bib-0014] Boonstra, L. D. (1953). A suggested clarification of the taxonomic status of the south African titanosuchians. Annals of the South African Museum, 42, 19–28.

[ar70038-bib-0015] Boonstra, L. D. (1962). The dentition of the titanosuchian dinocephalians. Annals of the South African Museum, 46, 57–112.

[ar70038-bib-0016] Boonstra, L. D. (1968). The braincase, basicranial axis and median septum in the Dinocephalia. Annals of the South African Museum, 50, 195–273.

[ar70038-bib-0017] Boonstra, L. D. (1969). The fauna of the *Tapinocephalus* zone (Beaufort beds of the Karoo). Annals of the South African Museum, 56, 1–73.

[ar70038-bib-0018] Brink, A. S. (1958). *Struthiocephalus kitchingi* sp. nov. Palaeontologia africana, 5, 39–56.

[ar70038-bib-0019] Brink, A. S. (1977). A model of tooth replacement in the ‘mammal‐like reptile’ *Diademodon* . South African Journal of Science, 73, 138–143.

[ar70038-bib-0020] Broom, R. (1932). The mammal‐like reptiles of South Africa and the origin of mammals. H.F. & G. Witherby.

[ar70038-bib-0021] Button, D. J. , Barrett, P. M. , & Rayfield, E. J. (2016). Comparative cranial myology and biomechanics of *Plateosaurus* and *Camarasaurus* and evolution of the sauropod feeding apparatus. Palaeontology, 59, 887–913. 10.1111/pala.12266

[ar70038-bib-0022] Button, D. J. , Barrett, P. M. , & Rayfield, E. J. (2017). Craniodental functional evolution in sauropodomorph dinosaurs. Paleobiology, 43, 435–462. 10.1017/pab.2017.4

[ar70038-bib-0023] Castanhinha, R. , Araújo, R. , Júnior, L. C. , Angielczyk, K. D. , Martins, G. G. , Martins, R. M. S. , Chaouiya, C. , Beckmann, F. , & Wilde, F. (2013). Bringing dicynodonts back to life: Paleobiology and anatomy of a new emydopoid genus from the upper Permian of Mozambique. PLoS One, 8, e80974. 10.1371/journal.pone.0080974 24324653 PMC3852158

[ar70038-bib-0024] Christiansen, P. (2000). Feeding mechanisms of the sauropod dinosaurs *Brachiosaurus*, *Camarasaurus*, *Diplodocus* and *Dicraeosaurus* . Historical Biology, 14, 137–152. 10.1080/10292380009380563

[ar70038-bib-0025] Codron, J. (2008). *Annals of ivory: Perspectives on African elephant* Loxodonta africana *Blumenbach 1797) feeding ecology from a multi‐decadal record*. Unpublished PhD thesis. University of Cape Town. Available from: http://hdl.handle.net/11427/4169

[ar70038-bib-0026] Cummins, F. , Reilly, P. O. , Flannery, O. , Kelly, D. , & Kenny, P. (2011). Defining the impaction frequency and threshold force required for femoral impaction grafting in revision hip arthroplasty: A human cadaveric mechanical study. Acta Orthopaedica, 82, 433–437. 10.3109/17453674.2011.594228 21689068 PMC3237033

[ar70038-bib-0027] Day, M. O. , & Rubidge, B. S. (2020). Biostratigraphy of the *Tapinocephalus* Assemblage Zone (Beaufort Group, Karoo Supergroup), South Africa. South African Journal of Geology, 123, 149–164. 10.25131/sajg.123.0012

[ar70038-bib-0028] Day, M. O. , & Rubidge, B. S. (2021). The late Capitanian mass extinction of terrestrial vertebrates in the Karoo Basin of South Africa. Frontiers in Earth Science, 9, 631198. 10.3389/feart.2021.631198

[ar70038-bib-0029] Dean, M. C. , & Lucas, V. S. (2009). Dental and skeletal growth in early fossil hominins. Annals of Human Biology, 36, 545–561. 10.1080/03014460902956725 19579096

[ar70038-bib-0030] D'Emic, M. D. , Finch, S. P. , Britt, B. B. , & Wilson Mantilla, J. A. (2024). Increased sampling reveals the complex evolution of sauropod dinosaur tooth replacement rates. Journal of Anatomy. 10.1111/joa.14169 PMC12397056 39706808

[ar70038-bib-0031] D'Emic, M. D. , Whitlock, J. A. , Smith, K. M. , Fisher, D. C. , & Wilson, J. A. (2013). Evolution of high tooth replacement rates in sauropod dinosaurs. PLoS One, 8, e69235. 10.1371/journal.pone.0069235 23874921 PMC3714237

[ar70038-bib-0032] Dudgeon, T. W. , & Evans, D. C. (2023). Calvarial suture interdigitation in hadrosaurids (Ornithischia: Ornithopoda): Perspectives through ontogeny and evolution. Evolution & Development, 25, 209–225. 10.1111/ede.12430 36896717

[ar70038-bib-0033] Duhamel, A. , Benoit, J. , Wynd, B. , Wright, A. M. , & Rubidge, B. (2024). Redescription of three basal anomodonts: A phylogenetic reassessment of the holotype of *Eodicynodon oelofseni* (NMQR 2913). Frontiers in Earth Science, 11, 1220341. 10.3389/feart.2023.1220341

[ar70038-bib-0034] Edmund, A. G. (1960). Tooth replacement phenomenon in lower vertebrates *Life sciences division* (Vol. 52, pp. 1–190). Royal Ontario Museum.

[ar70038-bib-0035] Edmund, A. G. (1962). Sequence and rate of tooth replacement in the Crocodilia *Life sciences division* (Vol. 56, pp. 1–42). Royal Ontario Museum.

[ar70038-bib-0036] Erickson, G. M. (1996a). Daily deposition of dentine in juvenile *Alligator* and assessment of tooth replacement rates using incremental line counts. Journal of Morphology, 228, 189–194. 10.1002/(SICI)1097-4687(199605)228:2<189::AID-JMOR7>3.0.CO;2-0 29852586

[ar70038-bib-0037] Erickson, G. M. (1996b). Incremental lines of von Ebner in dinosaurs and the assessment of tooth replacement rates using growth line counts. Proceedings of the National Academy of Sciences, 93, 14623–14627. 10.1073/pnas.93.25.14623 PMC26184 8962103

[ar70038-bib-0038] Finch, S. P. , & D'Emic, M. D. (2022). Evolution of amniote dentine apposition rates. Biology Letters, 18, 20220092. 10.1098/rsbl.2022.0092 35472282 PMC9042580

[ar70038-bib-0039] Fiorillo, A. R. (1998). Dental micro wear patterns of the sauropod dinosaurs *Camarasaurus* and *Diplodocus*: Evidence for resource partitioning in the late Jurassic of North America. Historical Biology, 13, 1–16. 10.1080/08912969809386568

[ar70038-bib-0040] Fourie, S. (1963). Tooth replacement in the gomphodont cynodont *Diademodon* . South African Journal of Science, 57, 211–213.

[ar70038-bib-0041] Fox, D. L. (2000). Growth increments in *Gomphotherium* tusks and implications for late Miocene climate change in North America. Palaeogeography, Palaeoclimatology, Palaeoecology, 156, 327–348. 10.1016/S0031-0182(99)00148-0

[ar70038-bib-0042] Fraser‐King, S. W. , Benoit, J. , Day, M. O. , & Rubidge, B. S. (2019). Cranial morphology and phylogenetic relationship of the enigmatic dinocephalian *Styracocephalus platyrhynchus* from the Karoo Supergroup, South Africa. Palaeontologia africana, 54, 14–29.

[ar70038-bib-0043] García, R. A. , & Zurriaguz, V. (2016). Histology of teeth and tooth attachment in titanosaurs (Dinosauria; Sauropoda). Cretaceous Research, 57, 248–256. 10.1016/j.cretres.2015.09.006

[ar70038-bib-0044] Geist, V. (1972). An ecological and behavioural explanation of mammalian characteristics, and their implication to therapsid evolution. Zeitschrift Für Säugetierkunde, 37, 1–15.

[ar70038-bib-0045] Goswami, A. , Flynn, J. J. , Ranivoharimanana, L. , & Wyss, A. R. (2005). Dental microwear in Triassic amniotes: Implications for paleoecology and masticatory mechanics. Journal of Vertebrate Paleontology, 25, 320–329. 10.1671/0272-4634(2005)025[0320:DMITAI]2.0.CO;2

[ar70038-bib-0046] Green, J. L. (2012). Bone and dental histology of Late Triassic dicynodonts from North America. In A. Chinsamy‐Turan (Ed.), Forerunners of mammals: radiation, histology and biology (pp. 179–196). Indiana University Press.

[ar70038-bib-0047] Gregory, W. K. (1926). The skeleton of *Moschops capensis* Broom, a dinocephalian reptile from the Permian of South Africa. Bulletin of the American Museum of Natural History, 56, 179–251.

[ar70038-bib-0048] Güven, S. , Rubidge, B. S. , & Abdala, F. (2013). Cranial morphology and taxonomy of South African Tapinocephalidae (Therapsida: Dinocephalia): The case of *Avenantia* and *Riebeeckosaurus* . Palaeontologia africana, 48, 24–33.

[ar70038-bib-0049] He, Y. , Makovicky, P. J. , Xu, X. , & You, H. (2018). High‐resolution computed tomographic analysis of tooth replacement pattern of the basal neoceratopsian *Liaoceratops yanzigouensis* informs ceratopsian dental evolution. Scientific Reports, 8, 5870. 10.1038/s41598-018-24283-5 29651146 PMC5897341

[ar70038-bib-0050] Hendrickx, C. , Abdala, F. , & Choiniere, J. N. (2016). Postcanine microstructure in *Cricodon metabolus*, a Middle Triassic gomphodont cynodont from south‐eastern Africa. Palaeontology, 59, 851–861. 10.1111/pala.12263

[ar70038-bib-0051] Hopson, J. A. (1971). Postcanine replacement in the gomphodont cynodont *Diademodon* . In D. M. Kermack & K. A. Kermack (Eds.), Early mammals (pp. 1–21). Academic Press.

[ar70038-bib-0052] Ivakhnenko, M. F. (2008). Cranial morphology and evolution of Permian Dinomorpha (Eotherapsida) of eastern Europe. Paleontological Journal, 42, 859–995. 10.1134/S0031030108090013

[ar70038-bib-0053] Jasinoski, S. C. , & Chinsamy, A. (2012). Mandibular histology and growth of the nonmammaliaform cynodont *Tritylodon* . Journal of Anatomy, 220, 564–579. 10.1111/j.1469-7580.2012.01494.x

[ar70038-bib-0054] Jasinoski, S. C. , & Chinsamy‐Turan, A. (2012). Biological inferences of the cranial microstructure of the dicynodonts *Oudenodon* and *Lystrosaurus* . In A. Chinsamy‐Turan (Ed.), Forerunners of mammals: radiation, histology and biology (pp. 149–176). Indiana University Press.

[ar70038-bib-0055] Jaslow, C. R. (1989). Sexual dimorphism of cranial suture complexity in wild sheep (*Ovis orientalis*). Zoological Journal of the Linnean Society, 95, 273–284. 10.1111/j.1096-3642.1989.tb02312.x

[ar70038-bib-0056] Kammerer, C. F. (2011). Systematics of the Anteosauria (Therapsida: Dinocephalia). Journal of Systematic Palaeontology, 9, 261–304. 10.1080/14772019.2010.492645

[ar70038-bib-0057] Kammerer, C. F. (2021). Elevated cranial sutural complexity in burrowing dicynodonts. Frontiers in Ecology and Evolution, 9, 674151. 10.3389/fevo.2021.674151

[ar70038-bib-0058] Kato, K. M. , Rega, E. A. , Sidor, C. A. , & Huttenlocker, A. K. (2020). Investigation of a bone lesion in a gorgonopsian (Synapsida) from the Permian of Zambia and periosteal reactions in fossil non‐mammalian tetrapods. Philosophical Transactions of the Royal Society B: Biological Sciences, 375, 20190144. 10.1098/rstb.2019.0144 PMC7017433 31928188

[ar70038-bib-0059] Kemp, T. S. (1982). Mammal‐like reptiles and the origin of mammals. Academic Press Inc.

[ar70038-bib-0060] Kemp, T. S. (2005). The origin and evolution of mammals. Oxford University Press.

[ar70038-bib-0061] Kemp, T. S. (2006). The origin and early radiation of the therapsid mammal‐like reptiles: A palaeobiological hypothesis. Journal of Evolutionary Biology, 19, 1231–1247. 10.1111/j.1420-9101.2005.01076.x 16780524

[ar70038-bib-0062] Kermack, K. A. (1956). Tooth replacement in the mammal‐like reptiles of the suborders Gorgonopsia and Therocephalia. Philosophical Transactions of the Royal Society of London. B, Biological Sciences, 240, 95–133. 10.1098/rstb.1956.0013

[ar70038-bib-0063] Kitchener, A. (1988). An analysis of the forces of fighting of the blackbuck (*Antilope cervicapra*) and the bighorn sheep (*Ovis canadensis*) and the mechanical design of the horn of bovids. Journal of Zoology, 214, 1–20. 10.1111/j.1469-7998.1988.tb04983.x

[ar70038-bib-0064] Koch, P. L. (1989). Paleobiology of Late Pleistocene mastodonts and mammaths from southern Michigan and western New York. Unpublished PhD dissertation. University of Michigan. Available from: https://hdl.handle.net/2027.42/162478

[ar70038-bib-0065] Köhler, M. , Herridge, V. , Nacarino‐Meneses, C. , Fortuny, J. , Moncunill‐Solé, B. , Rosso, A. , Sanfilippo, R. , Palombo, M. R. , & Moyà‐Solà, S. (2021). Palaeohistology reveals a slow pace of life for the dwarfed Sicilian elephant. Scientific Reports, 11, 22862. 10.1038/s41598-021-02192-4 34819557 PMC8613187

[ar70038-bib-0066] Koyabu, D. , Werneburg, I. , Morimoto, N. , Zollikofer, C. P. E. , Forasiepi, A. M. , Endo, H. , Kimura, J. , Ohdachi, S. D. , Truong Son, N. , & Sánchez‐Villagra, M. R. (2014). Mammalian skull heterochrony reveals modular evolution and a link between cranial development and brain size. Nature Communications, 5, 3625. 10.1038/ncomms4625 PMC3988809 24704703

[ar70038-bib-0067] LeBlanc, A. R. H. , Brink, K. S. , Whitney, M. R. , Abdala, F. , & Reisz, R. R. (2018). Dental ontogeny in extinct synapsids reveals a complex evolutionary history of the mammalian tooth attachment system. Proceedings of the Royal Society B, 285, 20181792. 10.1098/rspb.2018.1792 30404877 PMC6235047

[ar70038-bib-0068] LeBlanc, A. R. H. , & Reisz, R. R. (2015). Patterns of tooth development and replacement in captorhinid reptiles: A comparative approach for understanding the origin of multiple tooth rows. Journal of Vertebrate Paleontology, 35, e919928. 10.1080/02724634.2014.919928

[ar70038-bib-0069] Luckett, W. P. (1993). Ontogenetic staging of the mammalian dentition, and its value for assessment of homology and heterochrony. Journal of Mammalian Evolution, 1, 269–282. 10.1007/BF01041667

[ar70038-bib-0070] Maho, T. , Maho, S. , Scott, D. , & Reisz, R. R. (2022). Permian hypercarnivore suggests dental complexity among early amniotes. Nature Communications, 13, 4882. 10.1038/s41467-022-32621-5 PMC9391490 35986022

[ar70038-bib-0071] Mallon, J. C. , & Anderson, J. S. (2014). The functional and palaeoecological implications of tooth morphology and wear for the megaherbivorous dinosaurs from the Dinosaur Park Formation (upper Campanian) of Alberta, Canada. PLoS One, 9, e98605. 10.1371/journal.pone.0098605 24918431 PMC4053334

[ar70038-bib-0072] Mao, F.‐Y. , Zheng, X.‐T. , Wang, X.‐L. , Wang, Y.‐Q. , Bi, S.‐D. , & Meng, J. (2019). Evidence of diphyodonty and heterochrony for dental development in euharamiyidian mammals from Jurassic Yanliao biota. Vertebrata Palasiatica, 57, 51–76. 10.19615/j.cnki.1000-3118.180803

[ar70038-bib-0073] Martinelli, A. G. , & Bonaparte, J. F. (2011). Postcanine replacement in *Brasilodon* and *Brasilitherium* (Cynodontia, Probainognathia) and its bearing in cynodont evolution. In J. Calvo , J. Porfiri , B. Gonzales Riga , & D. Dos Santos (Eds.), Paleontología y Dinosaurios Desde América Latina (pp. 179–186). Universidad Nacional de Cuyo.

[ar70038-bib-0074] Mason, R. , Rubidge, B. , & Hancox, J. (2015). Terrestrial vertebrate colonisation and the Ecca‐Beafort boundary in the southeastern Main Karoo Basin, South Africa: Implications for the Permian basin evolution. South African Journal of Geology, 118, 145–156. 10.2113/gssajg.118.2.145

[ar70038-bib-0075] Modesto, S. P. , Rubidge, B. S. , De Klerk, W. J. , & Welman, J. (2001). A dinocephalian therapsid fauna on the Ecca‐Beaufort contact in the eastern Cape Province, South Africa. South African Journal of Science, 97, 161–163.

[ar70038-bib-0076] Nacarino‐Meneses, C. , Jannello, J. M. , & Chinsamy, A. (2025). Life history data derived from the dental histological analysis of *Giraffa camelopardalis*: Implications for the palaeohistology of extinct giraffids. Journal of Anatomy. 10.1111/joa.14191 PMC12397227 39846502

[ar70038-bib-0077] Neumann, S. (2020). Taxonomic revision of the short‐snouted tapinocephalid Dinocephalia (Amniota‐Therapsida)—the key to understanding Middle Permian tetrapod biodiversity. PhD Thesis. University of the Witwatersrand. Available from: https://hdl.handle.net/10539/31602

[ar70038-bib-0078] Norton, L. A. (2020). Tooth replacement patterns in Eutheriodontia (Synapsida, Therapsida) from the South African Karoo Supergroup. PhD Thesis. University of the Witwatersrand. Available from: https://hdl.handle.net/10539/30172

[ar70038-bib-0079] Norton, L. A. , Abdala, F. , & Benoit, J. (2023). Craniodental anatomy in Permian–Jurassic Cynodontia and Mammaliaformes (Synapsida, Therapsida) as a gateway to defining mammalian soft tissue and behavioural traits. Philosophical Transactions of the Royal Society B: Biological Sciences, 378, 20220084. 10.1098/rstb.2022.0084 PMC10184251 37183903

[ar70038-bib-0080] Norton, L. A. , Abdala, F. , Rubidge, B. S. , & Botha, J. (2020). Tooth replacement patterns in the Early Triassic epicynodont *Galesaurus planiceps* (Therapsida, Cynodontia). PLoS One, 15, e0243985. 10.1371/journal.pone.0243985 33378326 PMC7773207

[ar70038-bib-0081] Norton, L. A. , Abdala, F. , Rubidge, B. S. , & Botha, J. (2021). Tooth replacement in the non‐mammalian cynodont *Cynosaurus suppostus* (Therapsida) from the late Permian of South Africa. Journal of Vertebrate Paleontology, 41, e2001650. 10.1080/02724634.2021.2001650

[ar70038-bib-0082] Olroyd, S. L. , LeBlanc, A. R. H. , Araújo, R. , Angielczyk, K. D. , Duhamel, A. , Benoit, J. , & Amaral, M. (2021). Histology and μCT reveal the unique evolution and development of multiple tooth rows in the synapsid *Endothiodon* . Scientific Reports, 11, 16875. 10.1038/s41598-021-95993-6 34413357 PMC8377087

[ar70038-bib-0083] O'Meara, R. N. , Dirks, W. , & Martinelli, A. G. (2018). Enamel formation and growth in non‐mammalian cynodonts. Royal Society Open Science, 5, 172293. 10.1098/rsos.172293 29892415 PMC5990740

[ar70038-bib-0084] Osborn, J. W. (1972). On the biological improbablity of Zahnreihen as embryological units. Evolution, 26, 601–607. 10.1111/j.1558-5646.1972.tb01967.x 28563353

[ar70038-bib-0085] Pusch, L. C. , Kammerer, C. F. , & Fröbisch, J. (2019). Cranial anatomy of the early cynodont *Galesaurus planiceps* and the origin of the mammalian endocranial characters. Journal of Anatomy, 234, 592–621. 10.1111/joa.12958 30772942 PMC6481412

[ar70038-bib-0086] Pusch, L. C. , Kammerer, C. F. , & Fröbisch, J. (2024). The origin and evolution of Cynodontia (Synapsida, Therapsida): Reassessment of the phylogeny and systematics of the earliest members of this clade using 3D‐imaging technologies. The Anatomical Record, 307, 1634–1730. 10.1002/ar.25394 38444024

[ar70038-bib-0087] Pusch, L. C. , Ponstein, J. , Kammerer, C. F. , & Fröbisch, J. (2020). Novel endocranial data on the early therocephalian *Lycosuchus vanderrieti* underpin high character variability in early theriodont evolution. Frontiers in Ecology and Evolution, 7, 464. 10.3389/fevo.2019.00464

[ar70038-bib-0088] Rodrigues, P. G. , Ruf, I. , & Schultz, C. L. (2014). Study of a digital cranial endocast of the non‐mammaliaform cynodont *Brasilitherium riograndensis* (later Triassic, Brazil) and its relevance to the evolution of the mammalian brain. Paläontologische Zeitschrift, 88, 329–352. 10.1007/s12542-013-0200-6

[ar70038-bib-0089] Rubidge, B. S. (1991). A new primitive dinocephalian mammal‐like reptile from the Permian of southern Africa. Palaeontology, 34, 547–559.

[ar70038-bib-0090] Rubidge, B. S. , Sidor, C. A. , & Modesto, S. P. (2006). A new burnetiamorph (Therapsida: Biarmosuchia) from the middle Permian of South Africa. Journal of Paleontology, 80, 740–749. 10.1666/0022-3360(2006)80[740:ANBTBF]2.0.CO;2

[ar70038-bib-0091] Rutter, N. W. , Geist, V. , & Shackleton, D. M. (1972). A Bighorn sheep skull 9280 years old from British Columbia. Journal of Mammalogy, 53, 641–644. 10.2307/1379066

[ar70038-bib-0092] Ryan, A. S. (1981). Anterior dental microwear and its relationship to diet and feeding behavior in three African primates (*Pan troglodytes troglodytes*, *Gorilla gorilla gorilla* and *Papio hamadryas*). Primates, 22, 533–550. 10.1007/BF02381245

[ar70038-bib-0093] Salgado, L. , & Calvo, J. O. (1997). Evolution of titanosaurid sauropods. II: The cranial evidence. Ameghiniana, 34, 33–48.

[ar70038-bib-0094] Schour, I. , & Hoffman, M. M. (1939). Studies in tooth development: I. The 16 microns calcification rhythm in the enamel and dentin from fish to man. Journal of Dental Research, 18(1), 91–102. 10.1177/00220345390180010701

[ar70038-bib-0095] Sereno, P. C. , Wilson, J. A. , Witmer, L. M. , Whitlock, J. A. , Maga, A. , Ide, O. , & Rowe, T. A. (2007). Structural extremes in a Cretaceous dinosaur. PLoS One, 2, e1230. 10.1371/journal.pone.0001230 18030355 PMC2077925

[ar70038-bib-0096] Sidor, C. A. , & Welman, J. (2003). A second specimen of *Lemurosaurus pricei* (Therapsida: Burnetiamorpha). Journal of Vertebrate Paleontology, 23, 631–642. 10.1671/0272-4634(2003)023[0631:ASSOLP]2.0.CO;2

[ar70038-bib-0097] Smith, J. B. , & Dodson, P. (2003). A proposal for a standard terminology of anatomical notation and orientation in fossil vertebrate dentitions. Journal of Vertebrate Paleontology, 23, 1–12. 10.1671/0272-4634(2003)23[1:APFAST]2.0.CO;2

[ar70038-bib-0098] Tatarinov, L. P. (1976). Morphological evolution of the theriodonts and the general problems of phylogenetics. Nauka.

[ar70038-bib-0099] Teaford, M. F. , & Runestad, J. A. (1992). Dental microwear and diet in Venezuelan primates. American Journal of Physical Anthropology, 88, 347–364. 10.1002/ajpa.1330880308 1642321

[ar70038-bib-0100] Thackeray, J. F. (1991). Growth increments in teeth of *Diictodon* (Therapsida). Koedoe, 34(1), 7–11. 10.4102/koedoe.v34i1.408

[ar70038-bib-0101] Vega, C. S. , & Maisch, M. W. (2014). Pathological features in upper Permian and Middle Triassic dicynodonts (Synapsida, Therapsida). In C. F. Kammerer , K. D. Angielczyk , & J. Fröbisch (Eds.), Early evolutionary history of the Synapsida *Vertebrate paleobiology and paleoanthropology* (pp. 151–161). Springer Netherlands. 10.1007/978-94-007-6841-3_9

[ar70038-bib-0102] White, H. E. , Clavel, J. , Tucker, A. S. , & Goswami, A. (2020). A comparison of metrics for quantifying cranial suture complexity. Journal of the Royal Society Interface, 17, 20200476. 10.1098/rsif.2020.0476 33023399 PMC7653371

[ar70038-bib-0103] Whitlock, J. A. (2011). Inferences of diplodocoid (Sauropoda: Dinosauria) feeding behavior from snout shape and microwear analyses. PLoS One, 6, e18304. 10.1371/journal.pone.0018304 21494685 PMC3071828

[ar70038-bib-0104] Whitney, M. R. , & Sidor, C. A. (2019). Histological and developmental insights into the herbivorous dentition of tapinocephalid therapsids. PLoS One, 14, e0223860. 10.1371/journal.pone.0223860 31665173 PMC6821052

[ar70038-bib-0105] Whitney, M. R. , & Sidor, C. A. (2020). Evidence of torpor in the tusks of *Lystrosaurus* from the Early Triassic of Antarctica. Communications Biology, 3, 471. 10.1038/s42003-020-01207-6 32855434 PMC7453012

[ar70038-bib-0106] Whyte, I. J. , & Hall‐Martin, A. (2018). Growth charactersitics of tusks of elephants in Kruger National Park. Pachyderm, 59, 31–40. 10.69649/pachyderm.v59i.78

[ar70038-bib-0107] Young, M. T. , Rayfield, E. J. , Holliday, C. M. , Witmer, L. M. , Button, D. J. , Upchurch, P. , & Barrett, P. M. (2012). Cranial biomechanics of *Diplodocus* (Dinosauria, Sauropoda): Testing hypotheses of feeding behaviour in an extinct megaherbivore. Naturwissenschaften, 99, 637–643. 10.1007/s00114-012-0944-y 22790834

[ar70038-bib-0108] Zar, J. H. (2013). Biostatistical analysis (5th ed.). Pearson Education Limited.

[ar70038-bib-0109] Zimmerman, L. M. (2020). The reptilian perspective on vertebrate immunity: 10 years of progress. Journal of Experimental Biology, 223, jeb214171. 10.1242/jeb.214171 33154186

